# Dam deformation forecasting using SVM-DEGWO algorithm based on phase space reconstruction

**DOI:** 10.1371/journal.pone.0267434

**Published:** 2022-06-01

**Authors:** Mingjun Li, Jiangyang Pan, Yaolai Liu, Yazhou Wang, Wenchuan Zhang, Junxing Wang

**Affiliations:** 1 China Power Construction Group Zhongnan Survey Design & Research Institute Co., Ltd., Changsha, China; 2 College of Water Conservancy and Hydropower, Hohai University, Nanjing, China; 3 Institute of HydroEcology, State Key Laboratory of Water Resources and Hydropower Engineering Science, Wuhan University, Wuhan, China; 4 Chang Jiang Survey, Planning, Design and Research CO., LTD., Wuhan, China; Torrens University Australia, AUSTRALIA

## Abstract

A hybrid model integrating chaos theory, support vector machine (SVM) and the difference evolution grey wolf optimization (DEGWO) algorithm is developed to analyze and predict dam deformation. Firstly, the chaotic characteristics of the dam deformation time series will be identified, mainly using the Lyapunov exponent method, the correlation dimension method and the kolmogorov entropy method. Secondly, the hybrid model is established for dam deformation forecasting. Taking SVM as the core, the deformation time series is reconstructed in phase space to determine the input variables of SVM, and the GWO algorithm is improved to realize the optimization of SVM parameters. Prior to this, the effectiveness of DEGWO algorithm based on the fusion of the difference evolution (DE) and GWO algorithm has been verified by 15 sets of test functions in CEC 2005. Finally, take the actual monitoring displacement of Jinping I super-high arch dam as examples. The engineering application examples show that the PSR-SVM-DEGWO model established performs better in terms of fitting and prediction accuracy compared with existing models.

## 1. Introduction

Dam is one of the most important engineering measures to regulate the spatio-temporal distribution of water resources and rationally allocate water resources, and it is also the key component of flood control engineering system [1, 2]. The safety status of dam engineering not only directly affects the full utilization of the benefits of the hydropower station, but also affects the life and property safety of the downstream people, even the ecological environment and social stability [3].

As a comprehensive response to dam behavior, deformation is an important indicator to evaluate dams’ safety [4]. By analyzing the measured data of dam displacement in time, and then establishing the corresponding prediction model, the deformation behavior and development trend of the dam can be accurately identified, and most hidden dangers can be further discovered to avoid the occurrence of catastrophic accidents.

According to different construction methods, conventional dam deformation prediction models are mainly divided into statistical models, deterministic models and hybrid models [5]. However, conventional models are difficult to adapt to the complex nonlinear relationship between multi-factors and effect sizes, and the accuracy of model predictions is hard to guarantee. With the development of machine learning (ML) technology, ML-based models are widely used to explain the structural behavior of dams [6]. Moreover, the existing prediction models only consider the main influencing factors, such as water pressure, temperature and aging, but do not consider the chaotic components that may be included in the deformed time series, which further limits the improvement of fitting accuracy [7].

Chaos is an irregular random behavior with initial sensitivity and ergodicity. Some studies [8, 9] have shown that there is chaos in the measured displacement data of dams. The efficient extraction of the chaotic component contained in the observation data is of great practical significance for improving the accuracy of prediction models. Zhang [9] proposed a new method based on empirical mode decomposition and phase space reconstruction theory to analyze the time-varying characteristics of dams. Su [10] combined SVM with phase space reconstruction, wavelet analysis, particle swarm optimization and other methods to establish a dam deformation prediction model. Gu [11] reconstructed the phase plane of the trend effect component of the dam service performance change through chaos technology. Wei [12] considered the chaotic effect of the residual sequence and proposed a new dam deformation prediction model. Obviously, when the phase space reconstruction method is used to design the input variable, the machine learning algorithm shows high applicability in improving the prediction accuracy. However, models based on machine learning algorithms are highly dependent on the adjustment of parameters, which can easily affect the stability of the prediction results. Therefore, using different heuristic search algorithms in the training process has become a popular method.

SVM are widely used in dam behavior prediction because of their obvious advantages in solving low-sample high-dimensional nonlinear problems [13]. Ren [14] used SVM modeling to effectively capture the complex relationships in deformation prediction. Hu [15] established a deformation prediction model for high arch dams during the initial operation period based on a least square support vector machine. The SVM can transform the original nonlinear problem into a high-latitude linear problem through the kernel parameters, so it is well adapted to the nonlinear deformation prediction. However, the selection of the kernel parameters and penalty factors of SVM will directly affect its performance in dealing with nonlinear problems.

Grey Wolf Optimizer (GWO) is a new intelligent optimization algorithm proposed by Mirjalili with reference to the social hierarchy and hunting behavior of grey wolves [16]. The algorithm realizes intelligent optimization through the process of tracking, encircling, hunting, and attacking the grey wolf population [17]. Studies have shown that the GWO algorithm is better than the other evolutionary algorithm in terms of quality, speed and stability of the final solution [17–19]. However, the possibility of premature convergence reduces the probability of the algorithm finding the global optimum. The initial population is an important factor influencing the optimization performance of an intelligent algorithm, but it is difficult to guarantee the diversity of the initial population with conventional GWO algorithms. To solve this problem, a hybrid GWO algorithm (HGWO) is proposed. It uses a differential evolution (DE) algorithm to generate a richer initial population. Use the proposed DEGWO algorithm to optimize the optimal kernel parameters and penalty factors of SVMs.

Based on the identification of the chaotic characteristics of the dam deformation observation data, this study combines SVMs and other methods to establish a dam deformation prediction model. The structure of this article is as follows. Section 2 introduces a variety of methods to identify chaotic characteristics of time series. In Section 3, a new dam deformation prediction model is proposed on the basis of phase space reconstruction. An hybrid GWO algorithm based on the fusion of the DE and GWO algorithm is introduced to optimize the parameter settings of SVM. The performance of the DEGWO algorithm is tested through 6 sets of test functions in CEC2005. In Section 4, taking the measured displacement data of Jinping I super high arch dam as an example, the method proposed in this paper and other common methods are used to establish the deformation prediction models. The prediction accuracy of models are compared and analyzed. Section 5 summarizes the main conclusions reached here.

## 2. Identification of chaotic characteristics

Chaotic systems are deterministic and sensitive to initial conditions [8]. It is worth noting that it is neither random nor disordered. At present, the identification of chaotic characteristics of time series is mainly based on phase space reconstruction, which can obtain more hidden information by recovering the chaotic attractor in the so-called high-dimensional phase space. The Lyapunov exponent, Correlation dimension and Kolmogorov entropy of the singular attractor are calculated to correctly distinguish the chaotic system from the random system [20]. When the correlation dimension *D*_2_ exists at a certain value, the maximum Lyapunov exponent *λ*_max_ is greater than 0 and the Kolmogorov entropy *K*_2_ is a finite positive value, it can be judged that the time series has chaotic characteristics.

### 2.1. Phase space reconstruction

The reconstruction of the phase space is the basis for the quantitative analysis of chaotic time series, in which the embedding dimension and the delay coordinate are the two most critical parameters [21]. The time delay method is currently the most commonly used method. For univariate chaotic time series {*x*_*i*_,*i* = 1,2,⋯,*n*}.


$$y_{i}=(x_{i},x_{i+\tau },\cdots ,x_{i+(m-1)\tau }),i=1,2,\cdots ,n-(m-1)\tau$$ (1)

According to Takens Theorem, the appropriate choice of the embedding dimension *m* and the delay time *τ* can restore the dynamics properties of the original state space in the sense of topological equivalence.

#### 2.1.1. Delay time

The mutual information method [22] is introduced to determine the delay time *τ* of the measured displacement sequence of the dam, as shown below.

$$I(\tau )=\sum_{x_{i},x_{i+\tau }}P(x_{i},x_{i+\tau })\log_{2}\{\frac{P(x_{i},x_{i+\tau })}{P(x_{i})(x_{i+\tau })}\}$$ (2)

where *P*(*x*_*i*_) is the normalized distribution of *x*_*i*_, *P*(*x*_*i*_*+τ*) is the normalized distribution of *x*_*i*_*+τ*, *P*(*x*_*i*,_*x*_*i+τ*_) is the joint distribution of *x*_*i*_ and *x*_*i*_*+τ*. The time at which the first minimum point appears in the *τ*~*I*(*τ*) curve is often selected as optimal the delay time.

#### 2.1.2. Embedding dimension

The embedding dimension *m* is determined by the Cao method [23]. The distance *a*(*t*,*m*) between the phase point and the nearest neighbor point is shown below.


$$a(t,m)=\frac{\left\|y_{t}^{m+1}-y_{f}^{m+1}\right\|}{\left\|y_{t}^{m}-y_{f}^{m}\right\|}$$ (3)

Where *y*_*t*_^*m*+1^ is the *t*th vector in the reconstructed phase space with the embedding dimension *m*+1, and *y*_*f*_^*m*+1^ is the nearest neighbor to *y*_*t*_^*m*+1^. *y*_*f*_^*m*^ is the nearest neighbor to *y*_*t*_^*m*^ in the reconstructed phase space with the embedding dimension *m*.

The average value *E*(*m*) of *a*(*t*,*m*) is calculated as follows.


$$E(m)=\frac{1}{n-m\tau }\sum_{t=1}^{n-m\tau }a(t,m)$$ (4)

Then, the change of *E*(*m*) is as follows.


$$E_{1}(m)=\frac{E(m+1)}{E(m)}$$ (5)

When *m* > *m*_0_, if *E*_1_(*m*) no longer changes, *m*_0_ represents the minimum embedding dimension. In order to avoid the situation that the change of *E*_1_(*m*) is difficult to judge, the Cao method adds another definition.


$$E^{'}(m)=\frac{1}{n-m\tau }\sum_{t=1}^{n-m\tau }\left|y_{t}^{m+1}-y_{f}^{m+1}\right|$$ (6)

$$E_{2}(m)=\frac{E^{'}(m+1)}{E^{'}(m)}$$ (7)

For chaotic time series, there will always be some value of m, so that *E*_2_(*m*)≠1. By observing whether *E*_1_(*m*) tends to be stable and whether the value of *m* can achieve *E*_2_(*m*)≠1 to determine the minimum *m* of the reconstructed phase space.

### 2.2. Lyapunov exponent method

The maximum Lyapunov exponent (*λ*_max_) is usually regarded as an indicator of chaotic motion [24]. *λ*_max_ > 0 indicates that the system is in a chaotic state. The specific process of calculating *λ*_max_ by the wolf method is as follows.

For the initial point *Y*(*t*_0_) in the phase space, the distance between it and the nearest neighbor *Y*_0_(*t*_0_) is *L*(*t*_0_). As time evolves, when the distance between two points exceeds the specified value *ε*, that is

$$L^{'}(t_{1})=|Y(t_{1})-Y_{0}(t_{1})|>\varepsilon ,\varepsilon >0$$ (8)

Keep the point *Y*(*t*_1_), and find a point *Y*_1_(*t*_1_) near *Y*(*t*_1_) to ensure that ensure that the following conditions are met, that is

$$L(t_{1})=|Y(t_{1})-Y_{1}(t_{1})|<\varepsilon ,\varepsilon >0$$ (9)

And the angle between *L*’(*t*_1_) and *L*(*t*_1_) is as small as possible.

Record the total number of iterations *M* when *Y*(*t*) reaches the end of the time series, and the maximum Lyapunov exponent *λ*_max_ is calculated as follows.


$$\lambda_{\max}=\frac{1}{t_{M}-t_{0}}\sum_{k=1}^{M}\ln\frac{L^{'}(t_{k})}{L(t_{k-1})}$$ (10)

### 2.3. Correlation dimension method

The correlation dimension *D*_2_ is mainly determined by the Grassberger Procaccia algorithm [25]. Suppose *r* is the radius of the sphere centered on *y*_*i*_ and *y*_*j*_, then the correlation integral *C*(*r*) is given by:

$$C(r)=\underset{n\to \infty }{\lim}\frac{2}{n(n-1)}\sum_{i,j=1}^{n}\theta [r-\|y_{i}-y_{j}\|]$$ (11)

Where *θ*(⋅) is the Heaviside function:

$$\{\begin{array}{c} \theta (u)=\{\begin{array}{cc} 0, & u\leq 0\\ 1, & u>0 \end{array}\\ \underset{r\to 0}{\lim}C(r)\infty r^{D}(r\to 0) \end{array}$$ (12)

Where *D*_2_ is the correlation dimension

$$D_{2}=\log C_{n}(r)/\log r$$ (13)

Thus, draw log*C*_n_(*r*)/log*r* curve, and then the value of *D*_2_ can be determined according to the slope of the curve. As the embedding dimension *m* gradually increases, the slope of the curve converges, and the limit of convergence is the correlation dimension *D*_2_. The slope of the curve of a stochastic system will continue to increase with the increase of *d*, and there will be no convergence phenomenon.

### 2.4. Kolmogorov entropy method

Kolmogorov entropy [26] describes the generation rate of chaotic orbital information over time. Kolmogorov entropy reflects the chaos level of nonlinear dynamic systems, and the *K*_2_ entropy proposed by Grassberger and Procaccia is most commonly used as its estimate. *K*_2_ > 0 is a sufficient condition for the nonlinear system to be a chaotic system, and the *K*_2_ entropy can be estimated by the correlation integral method.


$$K_{2}=-\underset{r\to 0}{\lim}\;\underset{d\to \infty }{\lim}\frac{1}{\Delta m\tau }\log_{2}\frac{C_{m}(r)}{C_{m+\Delta m}(r)}$$ (14)

When the embedding dimension *m* is continuously increasing at intervals of Δ*m*, the stable estimation of Kolmogorov entropy can be obtained through the equal slope regression of Eq (12). It should be noted that the minimum value of *m* must be an integer greater than *D*_2_.

In a phase space with embedding dimension *i*, there is

$$x_{ij}={\left[\log_{2}(r)\right]}_{ij}$$ (15)

$$y_{ij}={\left[\log_{2}(C(r))\right]}_{ij}$$ (16)

Where:

$$y_{ij}=ax_{ij}+b$$ (17)

Let *a* = *D*_2_, and for the embedding dimensions *i* and *i* +Δ*m*, there is

$$K_{2}=\underset{i\to \infty }{\lim}\frac{\Delta b_{i}}{\Delta m\tau }$$ (18)

Where Δ*b* = *b*_*i*_-*b*_*i*+*m*_.

The Kolmogorov entropy estimate *K*_2_ can be used to judge the motion properties of the nonlinear system: *K*_2_ = 0 means the nonlinear system performs regular motion, *K*_2_ > 0 means the nonlinear system performs chaotic motion, and *K*_2_ < 0 means the nonlinear system performs random motion.

## 3. Chaotic time series prediction

For the dam deformation time series *x*_*i*_(*i* = 1,2,…,*n*), when the delay time *τ* and the embedding dimension *m* have been determined, the phase space reconstruction results of the series *x*_*i*_ are as follows.


$$X=[\begin{array}{ccccc} x_{1} & x_{1+\tau } & x_{1+2\tau } & \cdots & x_{1+(m-1)\tau }\\ x_{2} & x_{2+\tau } & x_{2+2\tau } & \cdots & x_{2+(m-1)\tau }\\ x_{3} & x_{3+\tau } & x_{3+2\tau } & \cdots & x_{3+(m-1)\tau }\\ \vdots & \vdots & \vdots & & \vdots \\ x_{n-(m-1)\tau } & x_{n-(m-1)\tau +\tau } & x_{n-(m-1)\tau +2\tau } & \cdots & x_{n} \end{array}],Y=[\begin{array}{c} x_{2+(m-1)\tau }\\ x_{3+(m-1)\tau }\\ \vdots \\ x_{n+1} \end{array}]$$ (19)

After determining the input variables and output variables of the model, a novel model based on phase space reconstruction for dam deformation predicting is proposed.

To clarify the influence of the reconstructed phase space as an input variable on the prediction performance of the model, a conventional model with dam deformation influence factors as input variables should be established. Early studies [3, 27, 28] have shown that water level, temperature and aging are the main factors affecting dam deformation, as shown below.


$$\left\{\begin{array}{l} H-H_{0},{\left(H-H_{0}\right)}^{2},{\left(H-H_{0}\right)}^{3},{\left(H-H_{0}\right)}^{4},\sin\frac{2\pi it}{365}-\sin\frac{2\pi it_{0}}{365},\cos\frac{2\pi it}{365}-\cos\frac{2\pi it_{0}}{365},\\ \sin\frac{4\pi it}{365}-\sin\frac{4\pi it_{0}}{365},\cos\frac{4\pi it}{365}-\cos\frac{4\pi it_{0}}{365},\theta -\theta_{0},\ln\theta -\ln\theta_{0} \end{array}\right\}$$ (20)

In order to match the consistency of the model and avoid the larger data information overwhelming the smaller data information, the input data of SVM is normalized. After completing the SVM training process, the output data of the SVM needs to be denormalized.

Normalization equation is as follows.


$$x_{i}^{'}=\frac{x_{i}-x_{\min}}{x_{\max}-x_{\min}}$$ (21)

Anti-normalization equation is as follows.


$$x_{i}=x_{i}^{'}(x_{\max}-x_{\min})+x_{\min}$$ (22)

Where, *x*_*i*_ is a sample data; *x*_*min*_ and *x*_*max*_ respectively represent the minimum and maximum sample data; *x*_*i*_^’^ is the normalized data.

The mean square error (MSE), mean absolute percentage error (MAPE) and square correlation coefficient (R^2^) are used to evaluate the performance of predictive models, as shown below [10].


$$MSE=\frac{1}{N}\sum_{i=1}^{N}{\left(y_{D}(i)-y(i)\right)}^{2}$$ (23)

$$\mathrm{MAPE}=\frac{1}{N}\sum_{i=1}^{N}\left|\frac{y_{D}(i)-y(i)}{y(i)}\right|$$ (24)

$$R^{2}=\frac{{\left(\sum_{i=1}^{N}\left(y_{D}(i)-{\bar{y}}_{D})(y(i)-\bar{y}\right)\right)}^{2}}{\sum_{i=1}^{N}{\left(y_{D}(i)-{\bar{y}}_{D}\right)}^{2}\sum_{i=1}^{N}{\left(y(i)-\bar{y}\right)}^{2}}$$ (25)

Where, *y*_*D*_ and ${\bar{y}}_{D}$ represent predicted values and predicted average values, *y* and $\bar{y}$ represent measured values and measured average values, and *N* represents the number of observed samples. The closer the R^2^ is to 1, the smaller the MSE and the MAPE, the better the prediction effect of the model.

### 3.1. Support vector machine

SVM [10] usually needs to establish a suitable function *f*(*x*) to describe the nonlinear relationship between the characteristic value *x*_*i*_ and the target value *y*_*i*_, as shown below.


$$f(x_{i})=w\cdot \varphi (x_{i})+b$$ (26)

Where, *w* is the coefficient vector, *φ*(*x*_*i*_) is the transformation function, *w* and *b* represent the weight and bias respectively. *w* and *b* are estimated by minimizing the regularized hazard function, as shown below

$$R(w)=\frac{1}{2}{\left\|w\right\|}^{2}+C\sum_{i=1}^{n}L_{\varepsilon }(y_{i},f(x_{i}))$$ (27)

Where:

$$L_{\varepsilon }(y_{i},f(x_{i}))=\max\{0,|y_{i}-f(x_{i})|-\varepsilon \}$$ (28)

Where, $\frac{1}{2}{\left\|w\right\|}^{2}$ is the regularization term, *C* is the penalty coefficient, and *L*_*ε*_(*y*_*i*_,*f*(*x*_*i*_)) is the *ε*-insensitive loss function.

The optimization object can be deducted as follows:

$$\min f(w,\xi^{-},\xi^{+})=\frac{1}{2}{\left\|w\right\|}^{2}+C\sum_{i=1}^{n}\left(\xi^{-},\xi^{+}\right)$$ (29)

Subject to

$$\{\begin{array}{c} y_{i}-[w\cdot \varphi (x_{i})]-b\leq \varepsilon +\xi^{-},\xi^{-}\geq 0\\ {}[w\cdot \varphi (x_{i})]+b-y_{i}\leq \varepsilon +\xi^{+},\xi^{+}\geq 0 \end{array}$$ (30)

Where, *ξ*^+^ and *ξ*^−^ represent slack variables.

The key is to establish the Lagrangian function.


$$\begin{array}{l} \max H(\partial_{i}^{-},\partial_{i}^{+})=-\frac{1}{2}\sum_{i=1}^{n}\sum_{j=1}^{n}\left(\partial_{i}^{-}-\partial_{i}^{+})(\partial_{j}^{-}-\partial_{j}^{+})K(x_{i},x_{j}\right)\\ \;+\sum_{i=1}^{n}y_{i}(\partial_{i}^{-}-\partial_{i}^{+})-\varepsilon \sum_{i=1}^{n}y_{i}(\partial_{i}^{-}+\partial_{i}^{+}) \end{array}$$ (31)

Subject to

$$\sum_{i=1}^{n}\left(\partial_{i}^{-}-\partial_{i}^{+}\right)=0,\partial_{i}^{-},\partial_{i}^{+}\in [0,C]$$ (32)

Therefore

$$f(x)=\sum_{i=1}^{n}\left(\partial_{i}^{-}-\partial_{i}^{+}\right)K(x_{i},x_{j})+b$$ (33)

Where *K*(*x*_*i*_,*x*_*j*_) represents kernel function, including polynomial, radial basis function and sigmoid etc.

### 3.2. Hybrid Grey Wolf Optimizer (HGWO, DEGWO)

Grey Wolf Optimizer (GWO) is a new intelligent optimization algorithm proposed by Mirjalili with reference to the social hierarchy and hunting behavior of gray wolves [29]. The GWO algorithm realizes the optimization of the intelligent algorithm through the process of tracking, encircling, hunting, and attacking the grey wolf population. The algorithm is characterized by simple principle, few adjustment parameters, easy implementation, and strong global search capability.

Many scholars have improved and applied research on the GWO algorithm from a specific perspective for specific problems. These improvements are mainly concentrated in the following aspects: (1) Improve the initial population for addressing the problem that the random generation method cannot guarantee the initial population diversity [30]. (2) Improve the search mechanism to keep the GWO algorithm away from the local optimum [31, 32]. (3) Adjust the way of parameter changes to balance the algorithm’s global and local search capabilities [33–35]. (4) Hybrid algorithm, which combines the advantages of multiple algorithms to improve the algorithm’s performance.

A hybrid GWO (DEGWO) algorithm is proposed, which uses a differential evolution (DE) algorithm to generate a richer initial population. The performance of the DEGWO algorithm is tested using 15 CEC2005 benchmark functions. The test results show that compared with the whale optimization algorithm (WOA), particle swarm optimization (PSO) algorithm and the original GWO algorithm, the DEGWO algorithm has higher execution efficiency.

#### 3.2.1. Grey Wolf Optimizer

Grey wolves [36, 37] have a very strict social dominance hierarchy, which is mainly divided into four parts: *α*,*β*,*δ* and *ω*. *α* is the best solution, followed by *β* and *δ*, and the remaining solutions belong to *ω*. The top three best wolves that are closest to their prey are *α*, *β* and *δ*, and they guide *ω* to search for prey in promising search areas. During the hunting process, the wolf will update its position around *α*, *β* and *δ*, as shown below.


$$\vec{D}=\vec{C}\cdot {\vec{X}}_{p(t)}-\vec{X}(t)$$ (34)

$$\vec{X}(t+1)={\vec{X}}_{p(t)}-\vec{A}\cdot \vec{D}$$ (35)

Where *t* is the current iteration number, ${\vec{X}}_{p(t)}$ is the current position of the prey, $\vec{X}(t)$ is the current position of the wolf, and $\vec{D}$ is the distance between the wolf and the prey.

The coefficient vectors $\vec{A}$ and $\vec{C}$ are as follows.


$$\vec{A}=2\vec{a}\cdot {\vec{r}}_{1}-\vec{a}$$ (36)

$$\vec{C}=2{\vec{r}}_{2}$$ (37)

Where ${\vec{r}}_{1}$ and ${\vec{r}}_{2}$ are two vectors randomly generated between [0, 1], and the convergence factor $\vec{a}$ linearly decreases from 2 to 0 in the iterative process.

Update the position of grey wolves.


$$\{\begin{array}{c} {\vec{D}}_{\alpha }={\vec{C}}_{1}\cdot {\vec{X}}_{\alpha }-\vec{X}\\ {\vec{D}}_{\beta }={\vec{C}}_{2}\cdot {\vec{X}}_{\beta }-\vec{X}\\ {\vec{D}}_{\delta }={\vec{C}}_{3}\cdot {\vec{X}}_{\delta }-\vec{X} \end{array}$$ (38)

$$\{\begin{array}{c} {\vec{X}}_{1}={\vec{X}}_{\alpha }-A_{1}\cdot ({\vec{D}}_{\alpha })\\ {\vec{X}}_{2}={\vec{X}}_{\beta }-A_{2}\cdot ({\vec{D}}_{\beta })\\ {\vec{X}}_{3}={\vec{X}}_{\delta }-A_{3}\cdot ({\vec{D}}_{\delta }) \end{array}$$ (39)

$$\vec{X}(t+1)=\frac{{\vec{X}}_{1}+{\vec{X}}_{2}+{\vec{X}}_{3}}{3}$$ (40)

Where ${\vec{X}}_{\alpha }$, ${\vec{X}}_{\beta }$, ${\vec{X}}_{\delta }$ represent the position of *α*,*β*,*δ* respectively, $\vec{X}$ represents the location of the current solution, ${\vec{C}}_{1}$, ${\vec{C}}_{2}$, and ${\vec{C}}_{3}$ represent randomly generated vectors, and ${\vec{D}}_{\alpha }$, ${\vec{D}}_{\beta }$, and ${\vec{D}}_{\delta }$ represent the distances of *α*, *β* and *δ* from other grey wolves, respectively. ${\vec{A}}_{1}$, ${\vec{A}}_{2}$ and ${\vec{A}}_{3}$ are random vectors, $\vec{X}(t+1)$ is the final position of *ω*.

#### 3.2.2. Differential evolution

Differential Evolution Algorithm (DE) is an efficient group-based heuristic search algorithm [38]. It mainly obtains the optimal solution through three operations of mutation, crossover and selection. The population size is *N*, *D* represents the dimensionality in the solution space, *x*_*i*_ = (*x*_1,*i*_, *x*_2,*i*_,*…*, *x*_*D*,*L*_) is the individual vector, and *G* = 0,1,*…*,*G*_max_ is the iteration time. $x_{i}^{G}=(x_{1,i}^{G},x_{2,i}^{G},\cdots ,x_{D,i}^{G})$ is the *i*-th individual in the *G*-th generation. $x_{L}=(x_{1,L},x_{2,L},\cdots ,x_{D,L})$ and $x_{U}=(x_{1,U},x_{2,U},\cdots ,x_{D,U})$ record the lower and upper limit of search space.

The initial population *P*_0_ is mainly randomly generated within the upper and lower limit (*x*_*L*_,*x*_*U*_), the *j*-th index of the *i*-th individual is obtained by Eq (39).


$$x_{j,i}^{0}=x_{j,L}+rand(0,1)\times (x_{1,U}-x_{j,L})$$ (41)

The mutation operation generates a new mutation vector $v_{i}^{G}$, as shown below.


$$v_{i}^{G}=x_{r_{1}}^{G}+F(x_{r_{2}}^{G}-x_{r_{3}}^{G})$$ (42)

Where $r_{1},r_{2},r_{3}\in \{1,2,\cdots ,N\}$ are randomly generated integers, and *F* is the magnification ratio of the control difference vector, which is a real number with a varying range between [0, 2].

The crossover operation is as follows:

$$u_{i}^{G}=\{\begin{array}{l} v_{j,i}^{G},if(rand_{j,i}\leq CR\;or\;j=j_{rand})\\ x_{j,i}^{G},otherwise \end{array}$$ (43)

Where *CR* is the cross probability, which takes a value between [0, 1].

Finally, the selection operation is performed. For specific problems, all mutation crossover individuals are evaluated. For specific problems, all mutation crossover individuals are evaluated. If the fitness of the current individual exceeds the previous generation, it means that the mutation crossover operation is successful, and the current individual is retained; if the current individual’s fitness is not as good as the previous generation, the better individual is retained. The individual with the optimal fitness will become the optimal value of this generation of individuals. When the termination condition is met, the evolution will stop, otherwise the next round will continue.

#### 3.2.3. Hybrid grey wolf optimization algorithm (HGWO, DEGWO)

**Fig 1:**
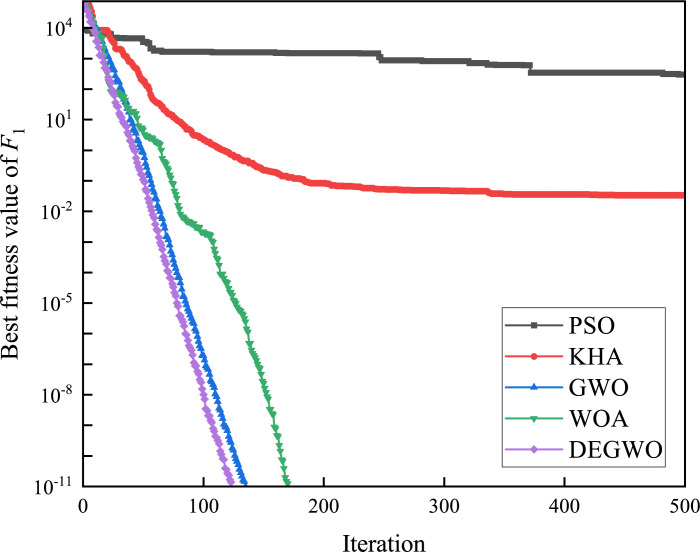
The pseudo code of DEGWO.

The conventional GWO algorithm randomly generates the initial population, which may fall into the dilemma of local optimization [39]. The DE algorithm generates group intelligence through mutual cooperation and competition between individuals. Based on the respective advantages and disadvantages of the GWO and DE algorithms, a more efficient hybrid algorithm (DE-GWO, HGWO) is proposed. The pseudo -code of the prosed DEGWO algorithm is shown in Fig 1. Specific steps are as follows.

1. Set the relevant parameters of the DEGWO algorithm, such as the population size *N*, the maximum number of iterations *t*_max_, the upper and lower limits of the search range *ub* and *lb*, etc.
2. Initialize the parameters *a*, *A* and *C*. Generate intermediates (variant populations) through evolutionary mutation operations, and then generate initial population individuals through competitive selection operations, and set the iteration time *t* = 1.
3. Calculate the objective function value of a single grey wolf individual and determine the best three individuals as *X*_*α*,_ *X*_*β*_ and *X*_*δ*_ respectively.
4. Calculate the distance between other grey wolf individuals and the optimal *X*_*α*,_ *X*_*β*_ and *X*_*δ*_ according to Eq (38), and update the position of each grey wolf according to Eq (39).
5. Update the values of *a*, *A* and *C*. Crossover and competition operations are applied to retain better individual positions and generate new individuals respectively.
6. Update the position of the first three grey wolves *X*_*α*,_ *X*_*β*_ and *X*_*δ*_.
7. Determine whether the maximum number of iterations *t*_max_ has been reached. If yes, exit and output the current objective function value of *X*_*α*_; otherwise, *t* = *t* + 1, and move to the third step to continue.

**Input**: the population size *N*, the maximum number of iterations *t*_max_, the upper and lower limits of the search range *ub* and *lb*, etc.

**Output**: The global optimum

**Begin**

    Initialize the parent population and offspring population

    Calculate the fitness of each agent by support vector machine (SVM)

    **X**_**α**_ = the best search agent

    X_β_ = the second search agent

    **X**_**γ**_ = the third best search agent

    **While** (*t*< *t*_max_)

        **For** each search agent

            Update the parent individual position of the current search agent by GWO algorithm

        **end for**

    Update *a*, *A* and *C*

    Produce mutant by Mutation of Differential evolution (DE)

    Cross mutant and produce an offspring population by Crossover of the DE algorithm

    Produce a new search agent

    Selection the better fitness

    Update the X_α_, X_β_, X_γ_ of the parent individual

    *t* = *t*+1

    **End while**

**Return** the global optimum

#### 3.2.4. Simulation experiment test

**Fig 2:**
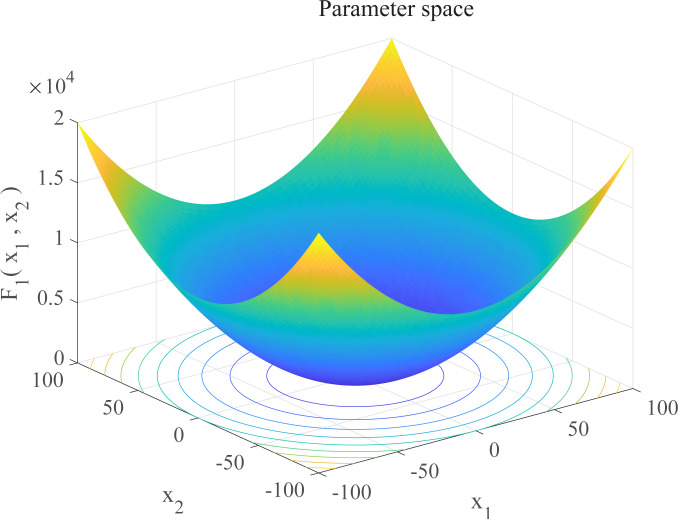
Three-dimensional graph of *F*_1_(*x*).

**Fig 3:**
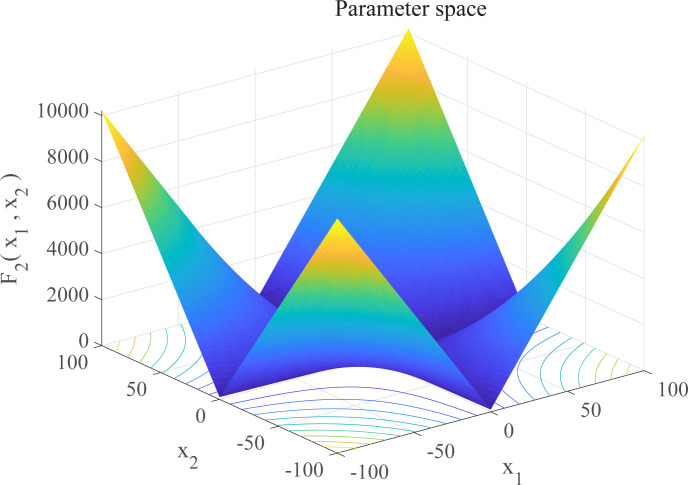
Three-dimensional graph of *F*_2_(*x*).

**Fig 4:**
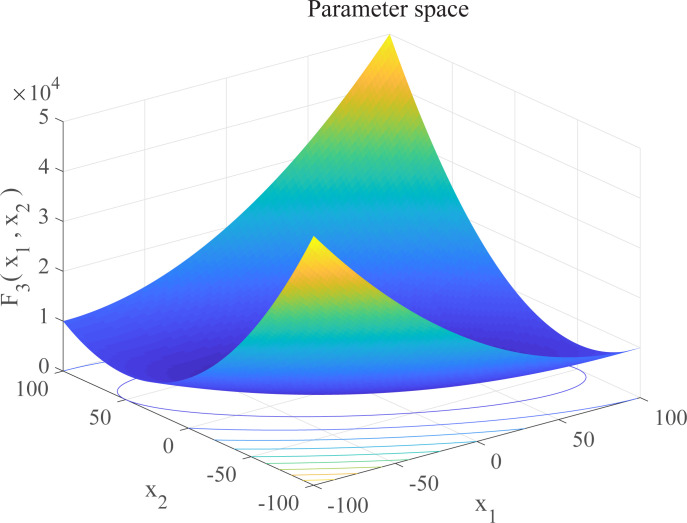
Three-dimensional graph of *F*_3_(*x*).

**Fig 5:**
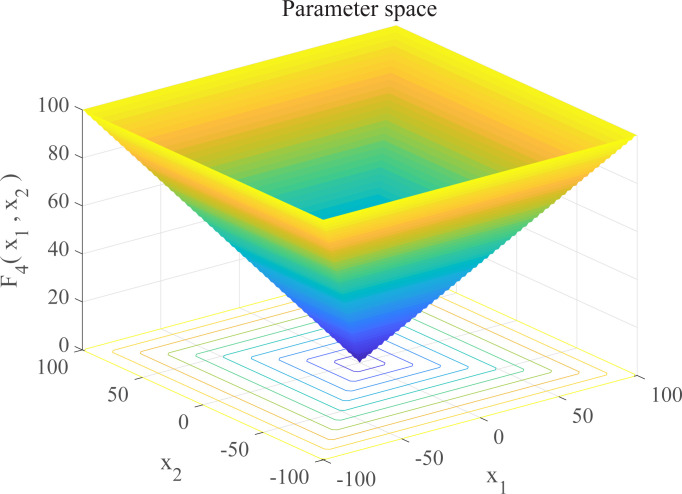
Three-dimensional graph of *F*_4_(*x*).

**Fig 6:**
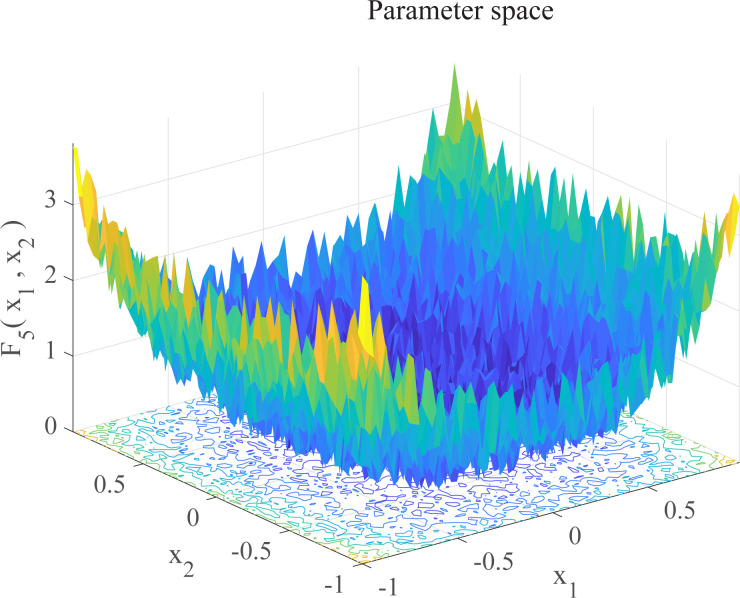
Three-dimensional graph of *F*_5_(*x*).

**Fig 7:**
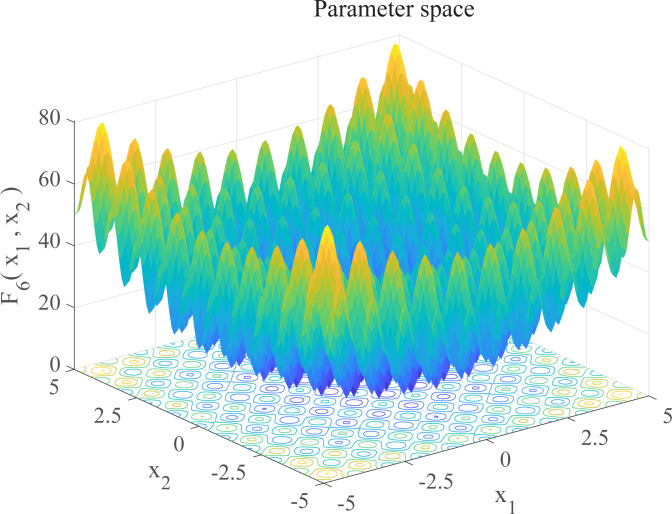
Three-dimensional graph of *F*_6_(*x*).

**Fig 8:**
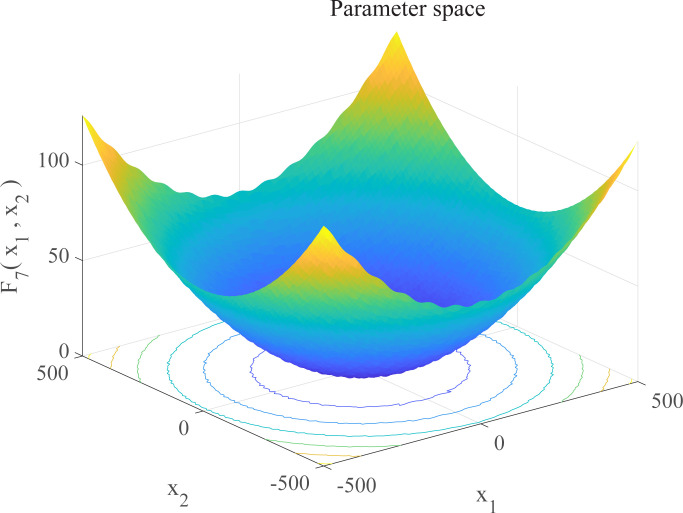
Three-dimensional graph of *F*_7_(*x*).

**Fig 9:**
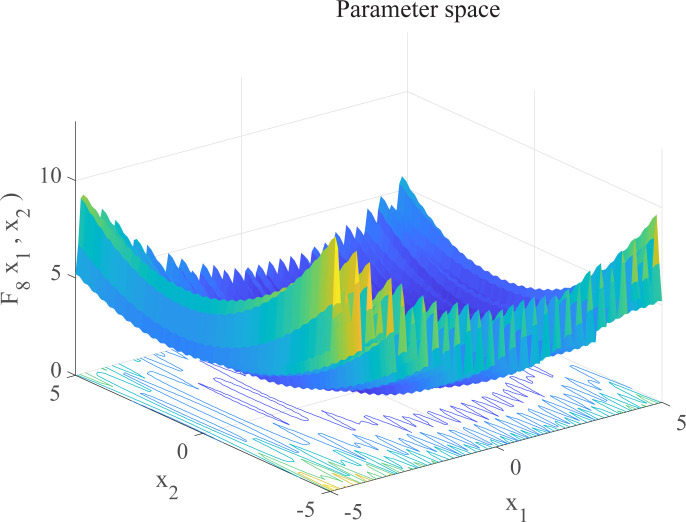
Three-dimensional graph of *F*_8_(*x*).

**Fig 10:**
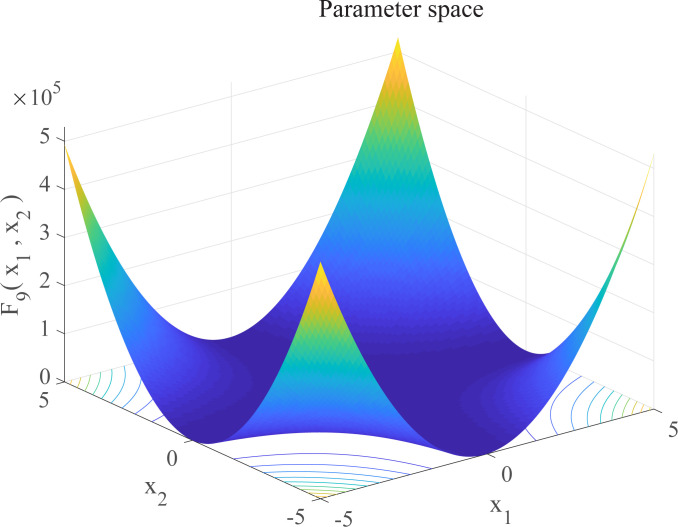
Three-dimensional graph of *F*_9_(*x*).

**Fig 11:**
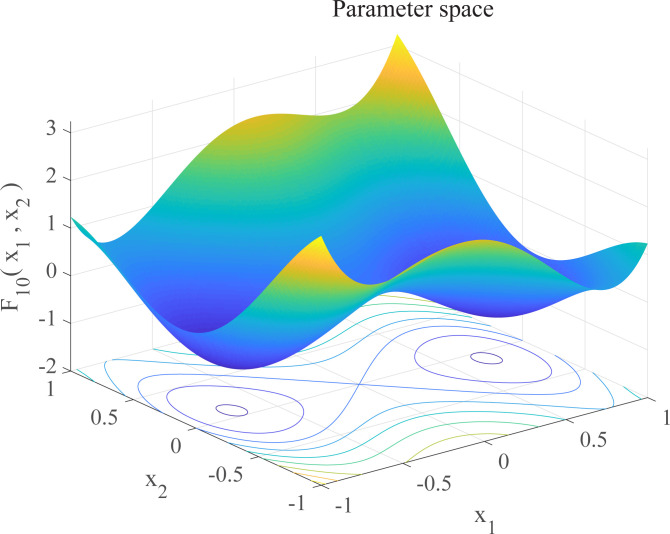
Three-dimensional graph of *F*_10_(*x*).

**Fig 12:**
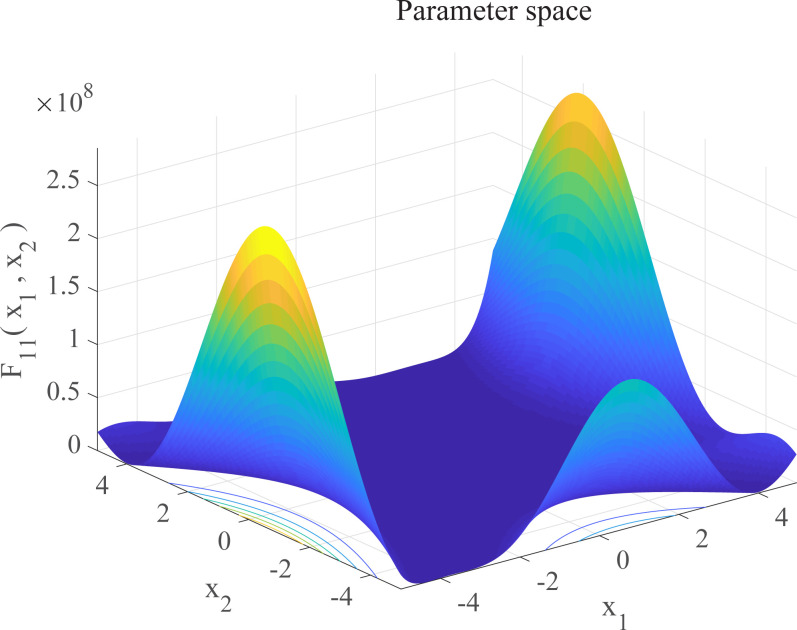
Three-dimensional graph of *F*_11_(*x*).

**Fig 13:**
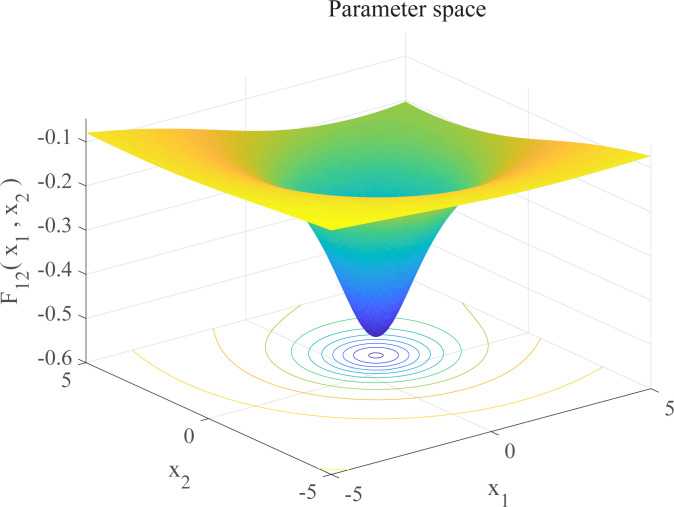
Three-dimensional graph of *F*_12_(*x*).

In this section, we use 12 sets of test functions in CEC 2005 [40] to prove the effectiveness of the DEGWO algorithm 5 groups of unimodal benchmark functions, 3 groups of multimodal benchmark functions and 4 groups of Fixed-dimension benchmark functions, as shown in Table 1 and Figs 2–13. The parameters of all intelligent algorithms are set as follows: population size *N* = 30, particle dimension *D* = 10, and maximum number of iterations *T* = 50. Note that the algorithm is run 20 times on each benchmark function.

**Table 1:** Benchmark functions.

	Function	Dim	Range	*f*_min_
Unimodal	$F_{1}(x)=\sum_{i=1}^{30}x_{i}^{2}$	30	[−100,100]	0
	$F_{2}(x)=\sum_{i=1}^{n}|x_{i}|+\prod_{i=1}^{n}\left|x_{i}\right|$	30	[−10,10]	0
	$F_{3}(x)={\sum_{i=1}^{n}\left(\sum_{j=1}^{i}x_{j}\right)}^{2}$	30	[−100,100]	0
	$F_{4}(x)=\max_{i}\{|x_{i}|,1\leq i\leq n\}$	30	[−100,100]	0
	$F_{5}(x)=\sum_{i=1}^{n}ix_{i}^{4}+random(0,1)$	30	[-1.28,1.28]	0
Multimodal	$F_{6}(x)=\sum_{i=1}^{n}\left[x_{i}{}^{2}-10\cos(2\pi x_{i})+10\right]$	30	[−5.12,5.12]	0
	$F_{7}(x)=\frac{1}{4000}\sum_{i=1}^{30}x_{i}{}^{2}-\prod_{i=1}^{30}\cos(\frac{x_{i}}{\sqrt{i}})+1$	30	[−600,600]	0
	$\begin{array}{l} F_{8}(x)=\\ 0.1\left\{\sin^{2}(3\pi x_{1})+\sum_{i=1}^{n}{\left(x_{i}-1\right)}^{2}[1+\sin^{2}(3\pi x_{i}+1)]+{\left(x_{n}-1\right)}^{2}[1+\sin^{2}(2\pi x_{n})]\right\}\\ +\sum_{i=1}^{n}u(x_{i},5,100,4)\\ y_{i}=1+\frac{x_{i}+1}{4}\\ u(x_{i},a,k,m)=\left\{ \begin{array}{l} k{\left(x_{i}-a\right)}^{m}\;x_{i}>a\\ \;0\;-a<x_{i}<a\\ k{\left(-x_{i}-a\right)}^{m}\;x_{i}<-a \end{array}\right. \end{array}$	30	[−50,50]	0
Fixed-dimension	$F_{9}(x)={\sum_{i=1}^{11}\left[a_{i}-\frac{x_{1}(b_{i}^{2}+b_{i}x_{2})}{b_{i}^{2}+b_{i}x_{3}+x_{4}}\right]}^{2}$	4	[−5,5]	0.00030
	$F_{10}(x)=4x_{1}^{2}-2.1x_{1}^{4}+\frac{1}{3}x_{1}^{6}+x_{1}x_{2}-4x_{2}^{2}+4x_{2}^{4}$	2	[−5,5]	−1.0316
	$\begin{array}{l} F_{11}(x)=\left[1+{\left(x_{1}+x_{2}+1\right)}^{2}(19-14x_{1}+3x_{1}^{2}-14x_{2}+6x_{1}x_{2}+3x_{2}^{2})\right]\times \\ \left[30+{\left(2x_{1}-3x_{2}\right)}^{2}\times (18-32x_{1}+12x_{1}^{2}+48x_{2}-36x_{1}x_{2}+27x_{2}^{2})\right] \end{array}$	2	[−2,2]	3
	$F_{12}(x)=‐{\sum_{i=1}^{10}\left[(X-a_{i}){\left(X-a_{i}\right)}^{T}+c_{i}\right]}^{-1}$	4	[0,10]	−10.5363

**Fig 14:**
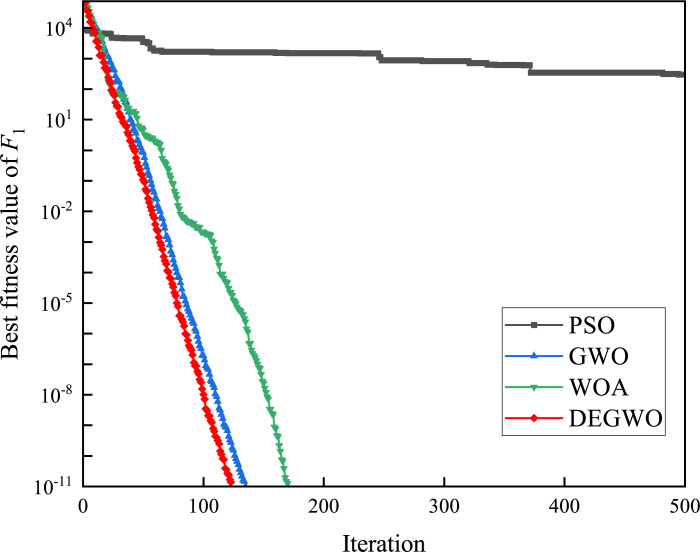
The convergence curves of *F*_1_(*x*).

**Fig 15:**
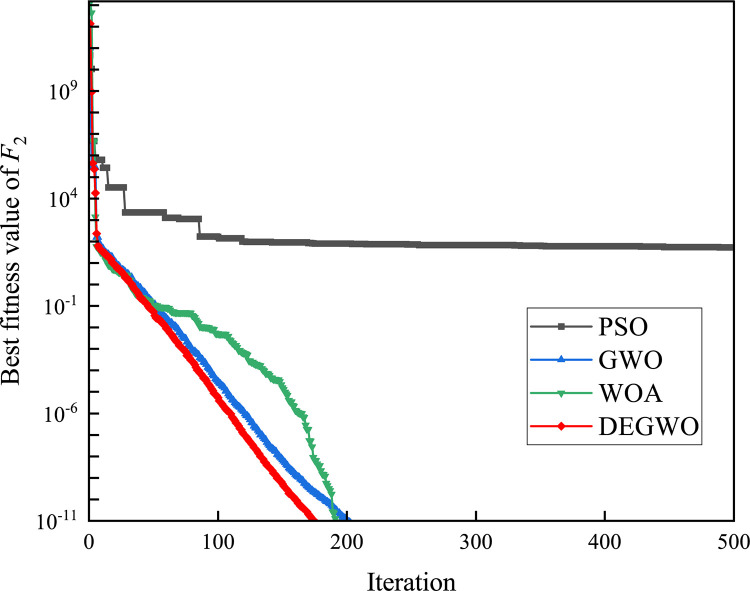
The convergence curves of *F*_2_(*x*).

**Fig 16:**
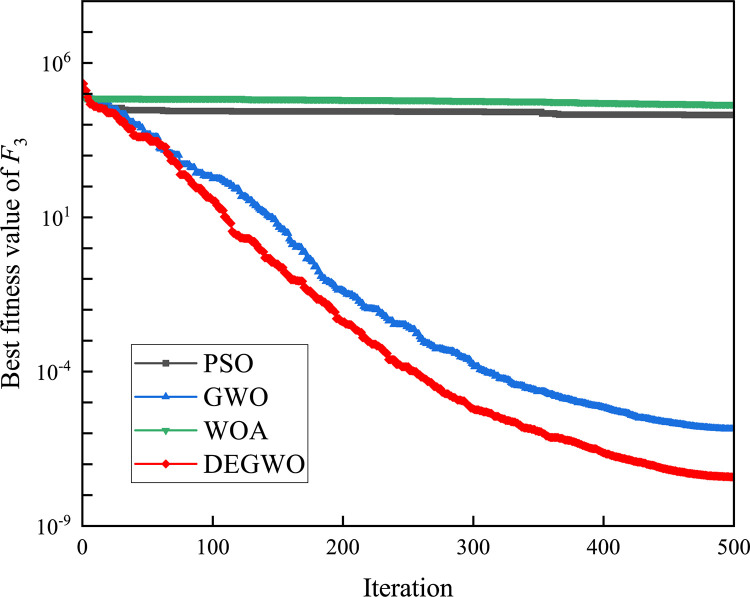
The convergence curves of *F*_3_(*x*).

**Fig 17:**
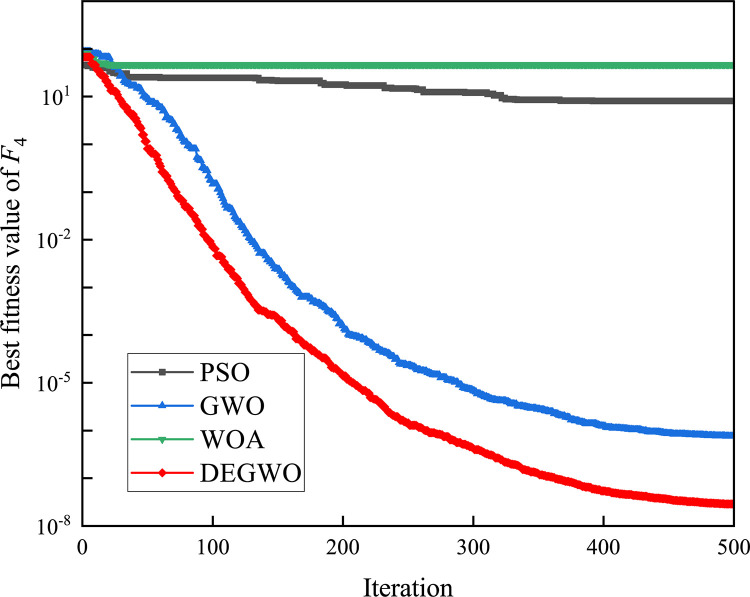
The convergence curves of *F*_4_(*x*).

**Fig 18:**
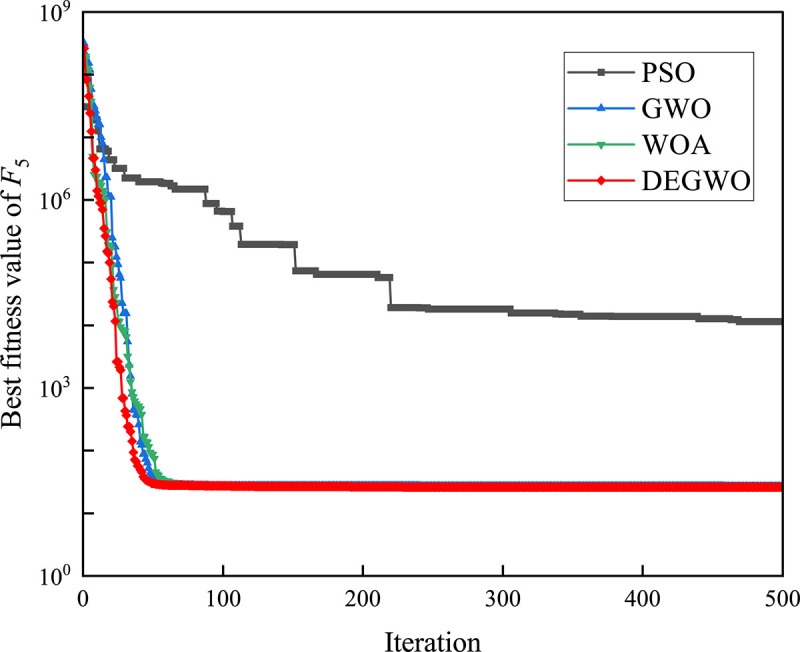
The convergence curves of *F*_5_(*x*).

**Fig 19:**
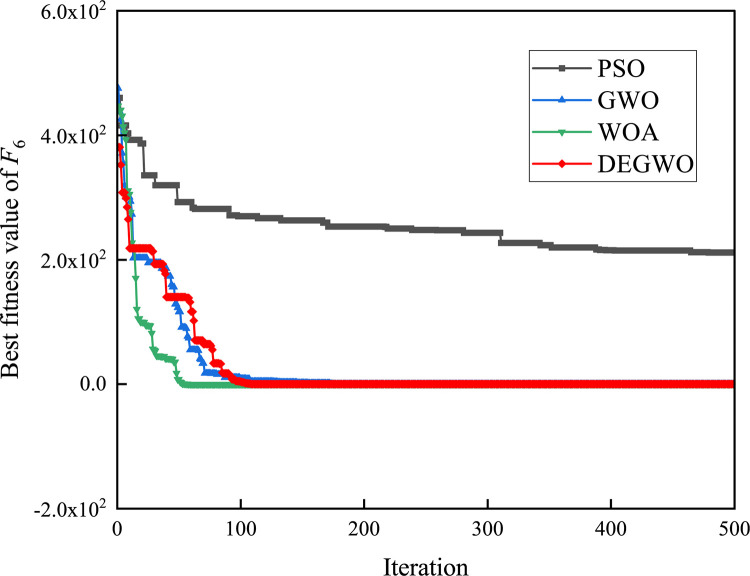
The convergence curves of *F*_6_(*x*).

**Fig 20:**
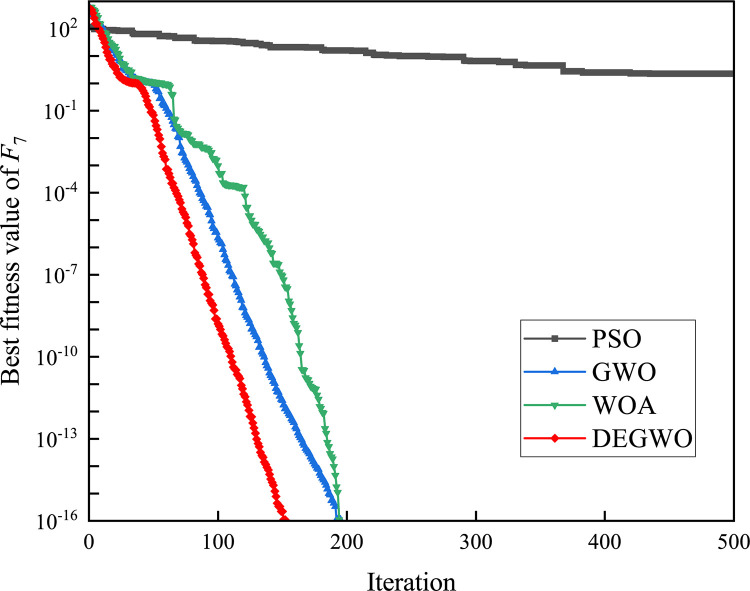
The convergence curves of *F*_7_(*x*).

**Fig 21:**
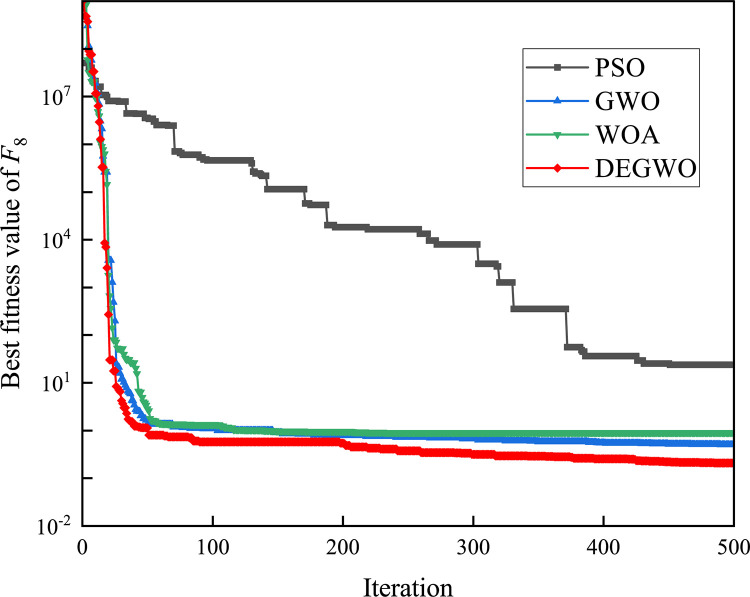
The convergence curves of *F*_8_(*x*).

**Fig 22:**
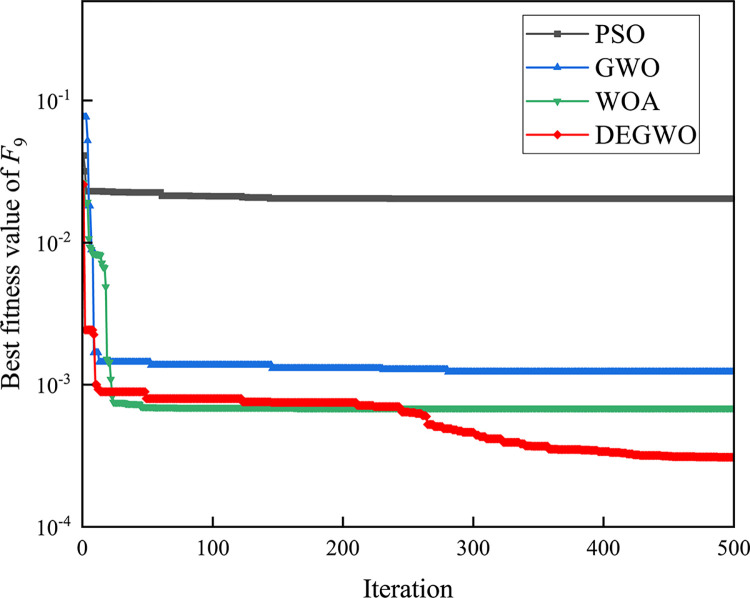
The convergence curves of *F*_9_(*x*).

**Fig 23:**
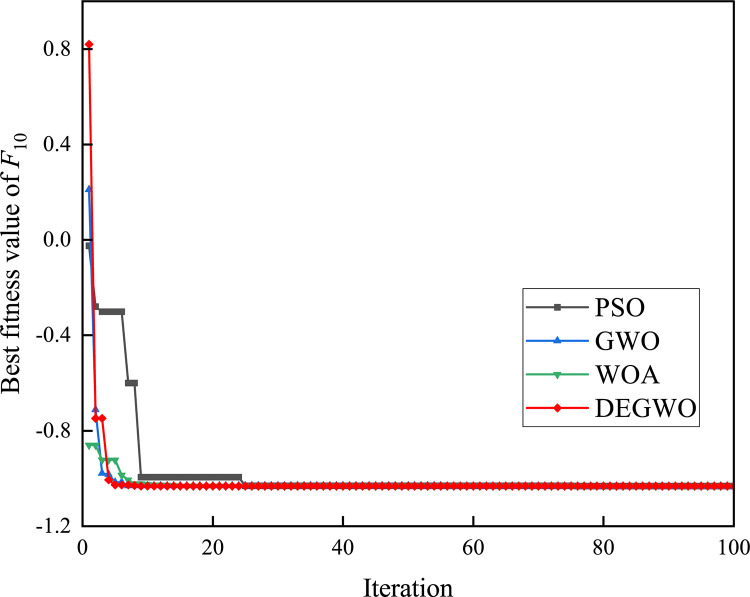
The convergence curves of *F*_10_(*x*).

**Fig 24:**
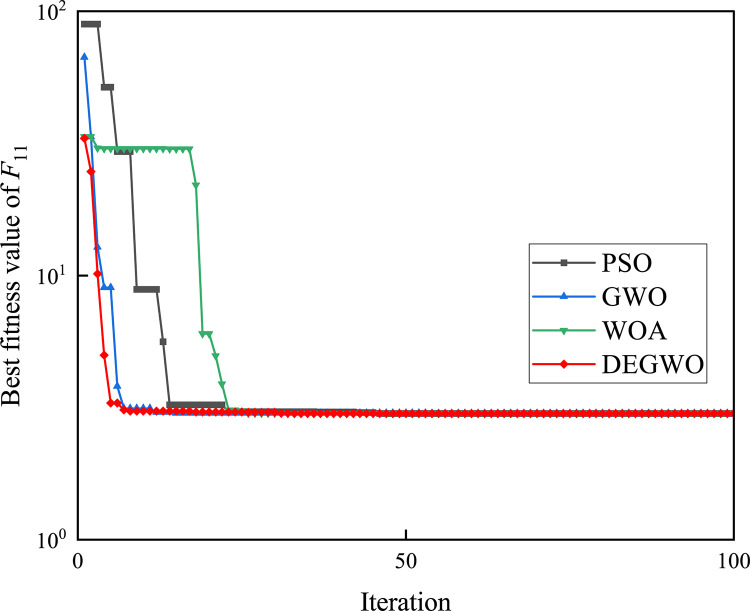
The convergence curves of *F*_11_(*x*).

**Fig 25:**
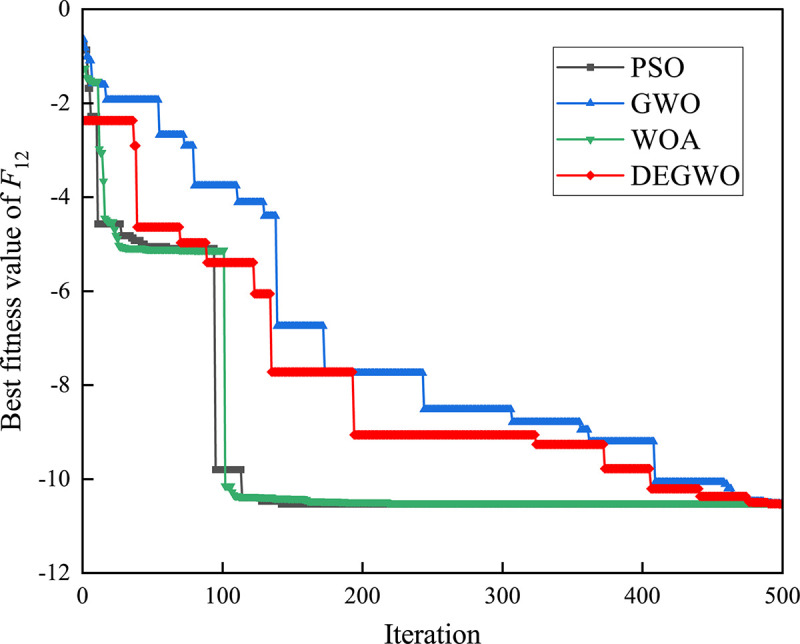
The convergence curves of *F*_12_(*x*).

Table 2 shows the final calculation results of PSO, GWO, WOA and DEGWO on the benchmark function. By comparing the average value (AVE) and standard deviation (STD), it is found that whether it is a single-peak benchmark function, a multi-peak benchmark function or a fixed-dimensional test function, the AVE and STD calculated by the DEGWO algorithm are in most cases smaller than those calculated by the other four algorithms. Figs 14–25 records the fitness curve changes of PSO, GWO, WOA and DEGWO algorithms in each test function, from which we can see the specific situation of each algorithm’s convergence. It can be seen from the downward trend of the curve in Figs 14–25 that the grey wolf individuals in the DEGWO algorithm can better update their positions as the number of iterations increases, and cooperate to achieve the purpose of improving the optimization results. Results show that compared with the other four algorithms, DEGWO has better convergence performance and can obtain more excellent solutions.

**Table 2:** Result of benchmark functions.

*F*	Index	PSO (1997)	GWO (2014)	WOA (2016)	DEGWO
***F***_**1**_	**AVE**	0.01200382	6.1124E-28	5.5358E-75	2.5427E-32
	**STD**	0.0376343	1.3289E-27	1.1575E-74	3.3545E-32
***F***_**2**_	**AVE**	3.13E+00	8.14E-17	1.22E-52	1.78E-19
	**STD**	4.4692	6.79E-17	1.86E-52	1.39E-19
***F***_**3**_	**AVE**	1.69E-01	1.28E-05	44044.7127	2.37E-08
	**STD**	0.236153	3.39E-05	12160.3861	2.10E-08
***F***_**4**_	**AVE**	4.11E-01	1.06E-06	41.7715	8.83E-08
	**STD**	0.418127	1.20E-06	24.8266	7.53E-08
***F***_**5**_	**AVE**	1.2326	2.17E-03	0.003207	1.89E-03
	**STD**	3.3498	1.59E-03	0.0038337	8.67E-04
***F***_**6**_	**AVE**	2.20E+02	-6177.811705	1.14E-14	-6284.556232
	**STD**	43.5764	559.991385	3.60E-14	717.5891418
***F***_**7**_	**AVE**	4.2883	0.0062066	0	0.0011031
	**STD**	2.0841	0.012158	0	0.0034883
***F***_**8**_	**AVE**	21.3245	0.72848	0.70863	0.38249
	**STD**	8.3245	0.27441	0.36232	0.17188
***F***_**9**_	**AVE**	0.013392	0.0024287	0.0012047	0.0044496
	**STD**	0.0099815	0.0063078	0.00058127	0.0083968
***F***_**10**_	**AVE**	-1.0315	-1.0316	-1.0316	-1.0316
	**STD**	0.00017899	4.10E-08	2.05E-10	2.88E-08
***F***_**11**_	**AVE**	3.0006	3	3	3
	**STD**	0.00077526	5.25E-05	4.08E-05	2.47E-05
***F***_**12**_	**AVE**	-10.5042	-10.5345	-8.369	-10.535
	**STD**	0.03792	0.0010846	2.986	0.00054846

### 3.3. Construction process of PSR-SVM-DEGWO model predicting dam deformation

**Fig 26:**
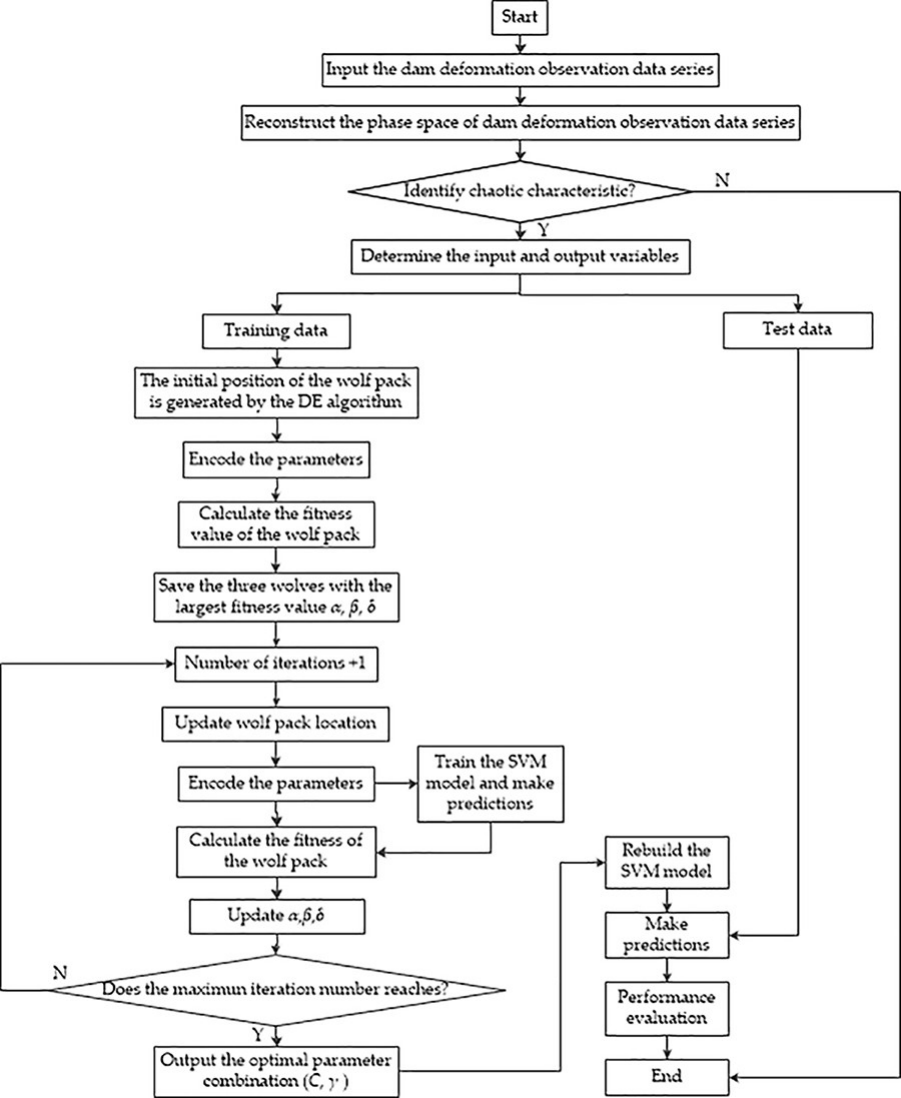
Construction process of dam deformation prediction model based on PSR-SVM-DEGWO.

A hybrid model combining chaos theory and SVM is proposed, and the DEGWO algorithm is used to select optimal parameters for concrete dam deformation analysis and prediction. Use the method mentioned in Section 2 to identify the chaotic characteristics of the deformed time series, and then determine the input variables of the support vector machine. According to the DEGWO algorithm introduced in Section 3.2, SVM is optimized for parameters. By performing training operations, a PSR-SVM-DEGWO prediction model can be established. The specific process is shown in Fig 26.

1. Reconstruct the phase space of the observed displacement time series. The mutual information method and the Cao method are adopted to determine the optimal delay time *τ* and the minimum embedding dimension *m*, and the phase space is reconstructed accordingly.
2. Identification of chaotic characteristics of the observed displacement time series. Estimate the maximum Lyapunov exponent *λ*_max_, correlation dimension *D*_2_ and Kolmogorov entropy *K*_2_. *λ*_max_ > 0, *K*_2_ is a finite positive value and the saturation of the correlation dimension indicating that the observed displacement time series has the chaotic characteristics.
3. Determine the input and output variables according to Eq (19).
4. Use the DEGWO algorithm to find the optimal SVM parameters based on the training samples, generate the optimal values of *C* and *σ*, and complete the SVM-DEGWO training process.
5. According to the prediction samples, the trained SVM will be used for prediction, and the three indicators of Eqs (23)–(25) are used to evaluate the prediction effect of the model.

## 4. Case study

### 4.1. Engineering overview

**Fig 27:**
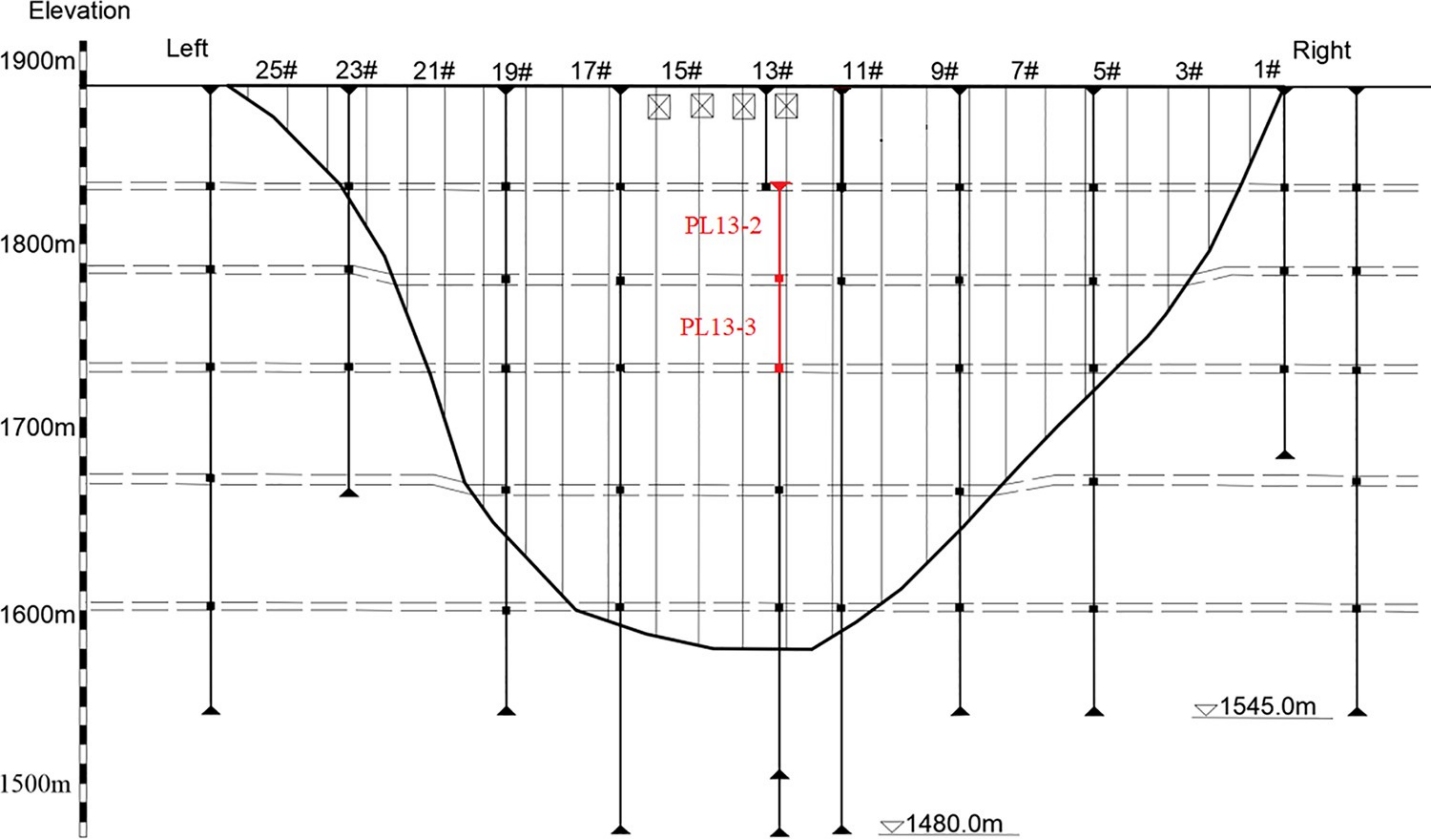
Vertical arrangement of the dam body.

The Jinping I Dam [41] is a concrete arch dam located in Sichuan Province, China. It is the highest arch dam in the world, and the maximum height of the dam is 305.0 m. The normal water storage level is 1880.0 m, and the dead water level is 1800.0 m. The layout of the dam vertical line monitoring system is shown in Fig 27.

**Fig 28:**
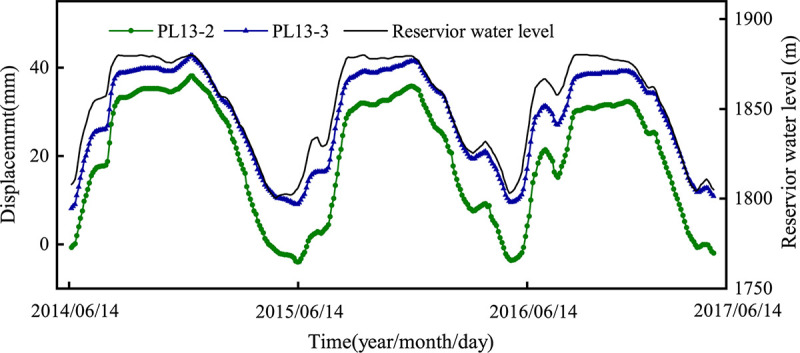
The observed horizontal displacement and water level of the measured points.

The monitoring points PL13-2 and PL13-3 are taken as examples. From June 2014 to June 2017, the horizontal displacement and water level changes of the measuring point are shown in Fig 28. Take the observation data from June 2014 to October 2017 as the training set, and the rest as the prediction set. As shown in Fig 28, the symbol (-) indicates the displacement to the downstream, and the symbol (+) indicates the displacement to the upstream. During the study period, the water level change range is [1750m,1900m], and the displacement showed obvious periodic changes with the water level change, and the change range is [-5mm,45mm].

### 4.2. Prediction model construction

**Fig 29:**
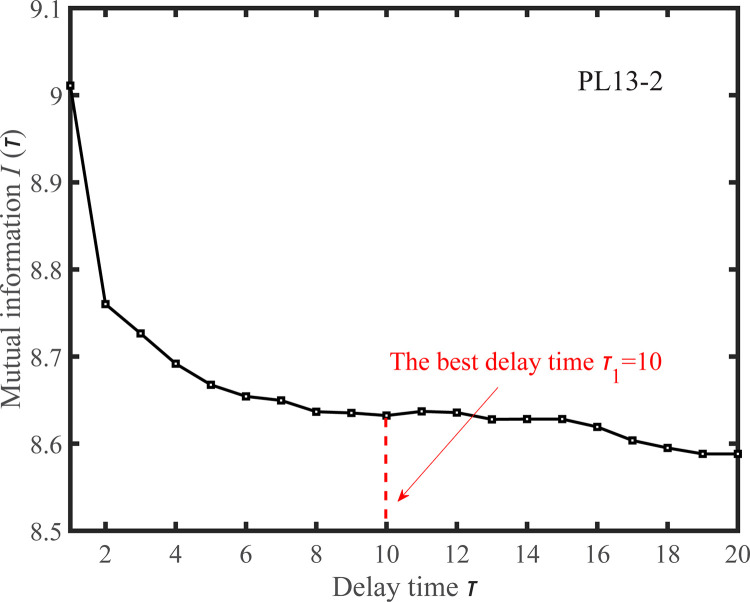
*I*(*τ*) ~ *τ* curve for the observed displacement data series of the PL13-2.

**Fig 30:**
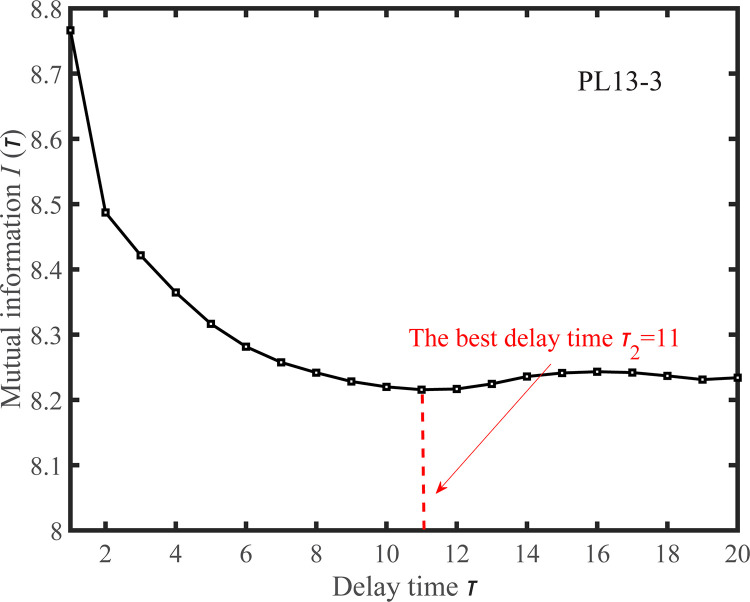
*I*(*τ*) ~ *τ* curve for the observed displacement data series of the PL13-3.

The delay time *τ* of the observed displacement time series is estimated by the mutual information method, as shown in Figs 29 and 30. According to the principle of mutual information, the optimal delay times of the measuring points PL13-2 and PL13-3 are *τ*_1_ = 10 and *τ*_2_ = 11, respectively.

**Fig 31:**
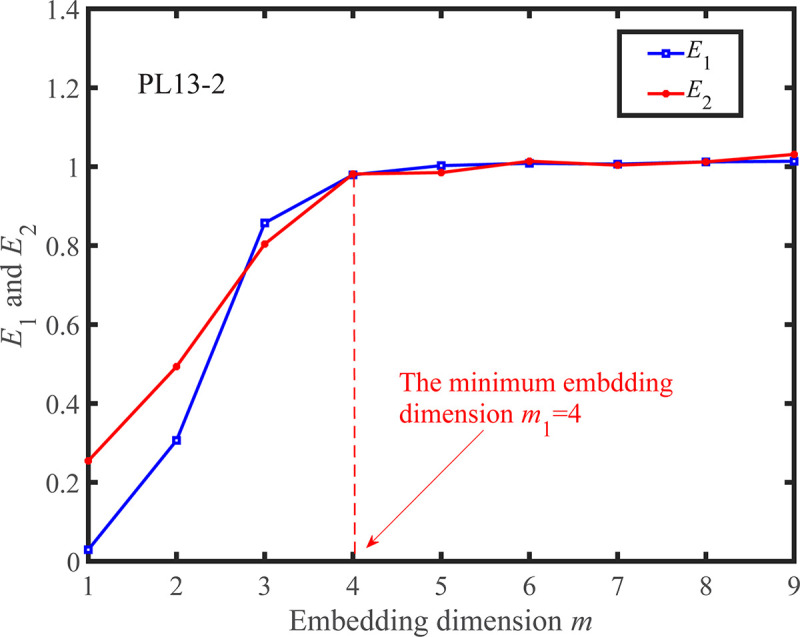
*E*_1_(*m*)~*m* and *E*_2_(*m*)~*m* curves for the observed displacement data series of the PL13-2.

**Fig 32:**
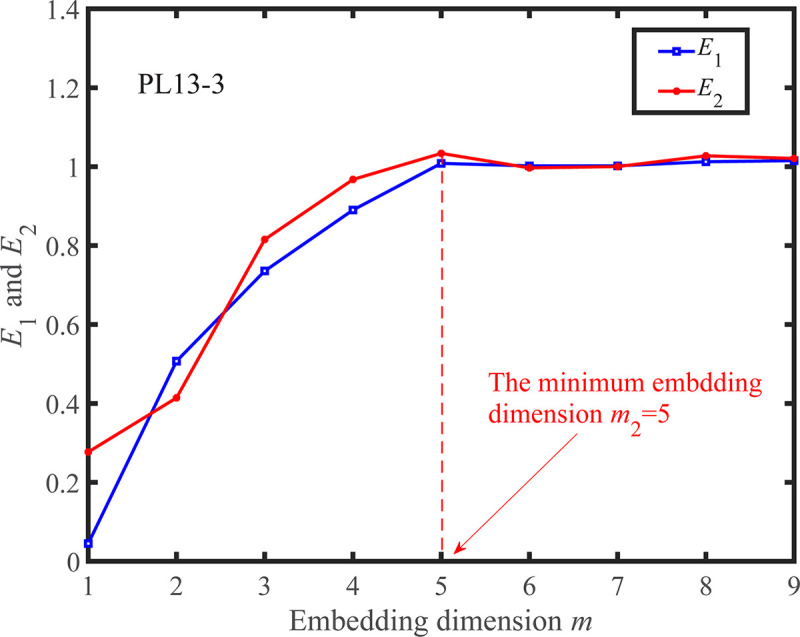
*E*_1_(*m*)~*m* and *E*_2_(*m*)~*m* curves for the observed displacement data series of the PL13-3.

According to the determined *τ*, the Cao method is adopted to calculate *m*. The change curves of *E*_1_(*m*) and *E*_2_(*m*) as *m* increases are shown in Figs 31 and 32. When *m* is greater than the minimum embedding dimension, the change of *E*_1_(*m*) starts to become smaller, and thus the optimal embedding dimension is determined. As shown in Figs 31 and 32, the minimum embedding dimension of the measuring points PL13-2, and PL13-3 are *m*_1_ = 4 and *m*_2_ = 5, respectively. It can also be found that there are some values of *m* such that *E*_2_(*m*)≠1, which can prove that the observed displacement data series comes from a deterministic process.

**Fig 33:**
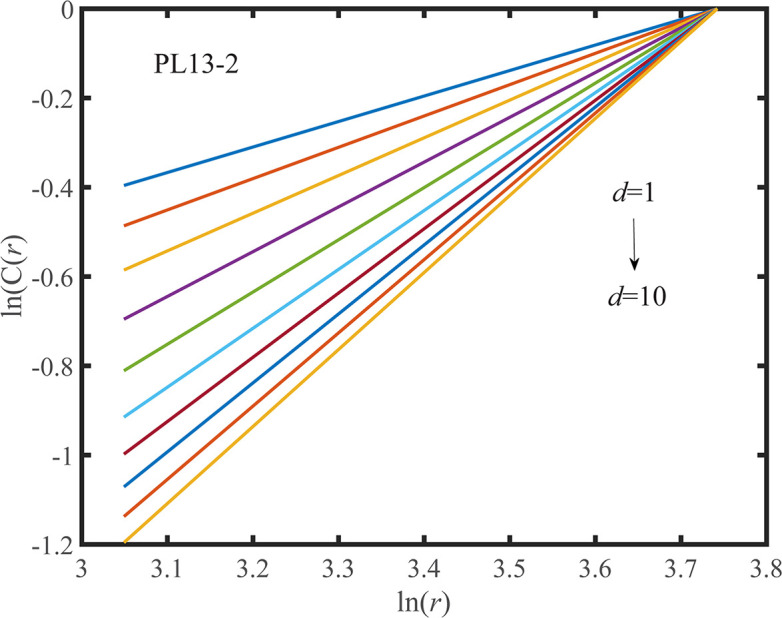
Double logarithmic curve of correlation dimension of the PL13-2.

**Fig 34:**
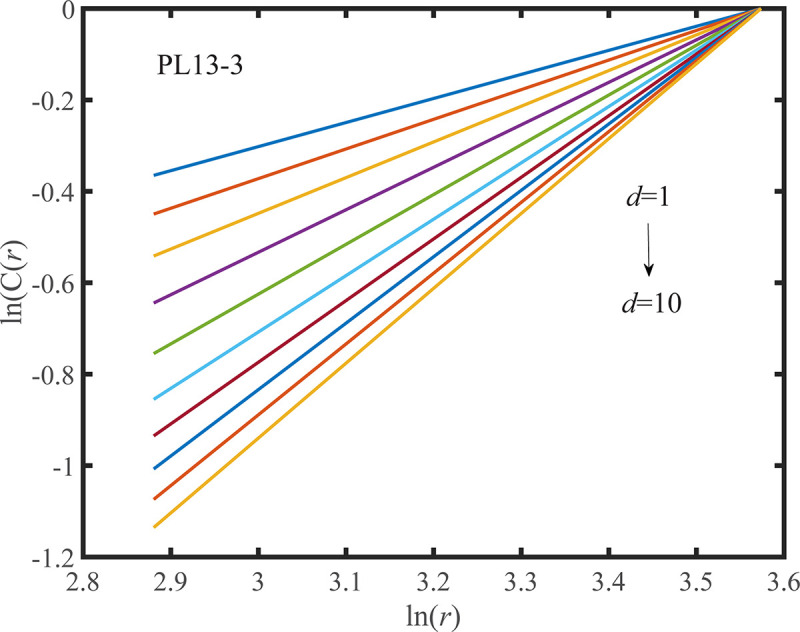
Double logarithmic curve of correlation dimension of the PL13-3.

According to the determined *τ*, the G-P method is adopted to calculate the correlation dimension of the observed displacement data series. When the value of the embedding dimension *d* ranges from 1 to 10, the corresponding double logarithmic curve of ln*C*(*r*)~ln(*r*) is shown in Figs 33 and 34. As shown in Figs 33 and 34 that the correlation index is saturated to a constant. According to the definition, it is concluded that the observed displacement data series is a chaotic series. And the correlation dimension of the measuring points PL13-2, and PL13-3 are *D*_2-1_ = 1.2043 and *D*_2-2_ = 1.1217, respectively.

The wolf method is adopted to calculate the maximum Lyapunov exponent *λ*_max_ of the observed displacement data series. And the maximum Lyapunov exponent λ_max_ of the measuring points PL13-2, and PL13-3 are *λ*_max−2_ = 0.0191 and *λ*_max−3_ = 0.0108, respectively. *λ*_max_ > 0 indicates that the observed displacement data series has chaotic characteristics.

The correlation integral method is used to calculate the Kolmogorov entropy estimate *K*_2_ of the observed displacement data series. According to the double logarithmic curve of ln*C*(*r*)~ln(*r*), as shown in Figs 33 and 34, the double logarithmic curve of log_2_(*r*)~log_2_(C(*r*)) can be derived, so that the Kolmogorov entropy estimate *K*_2_ of the measuring points PL13-2 and PL13-3 can be obtained when the delay time is determined respectively, and *K*_2-2_ = 0.0043, *K*_2-3_ = 0.0044. *K*_2_ takes a finite positive value, which indicates the chaotic characteristics of the observed displacement data series.

Based on the above, it can be concluded that the observed displacement data series of the measuring point PL13-2 and PL13-3 has chaotic characteristics, and a chaotic prediction model of dam displacement can be established.

The DEGWO algorithm proposed is adopted to seek the optimal parameters of SVM. The phase space of reconstructed observation displacement and the influencing factors of dam deformation are used as input variables to evaluate the predicting performance of the DEGWO and GWO algorithms. The population size *N* = 20, the maximum number of iterations *t*_max_ = 200, ε = 0.01, the penalty factor *C*∈[0.01,100], and the kernel parameter *γ*∈[0.01,1000]. The calculation is terminated when the number of iterations reaches 200.

**Fig 35:**
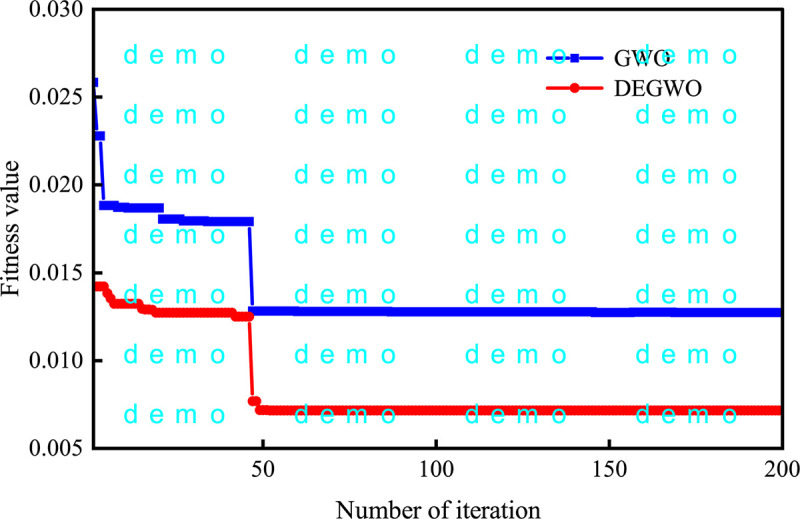
The fitness curves of the GWO and DEGWO model (conventional) of the PL13-2.

**Fig 36:**
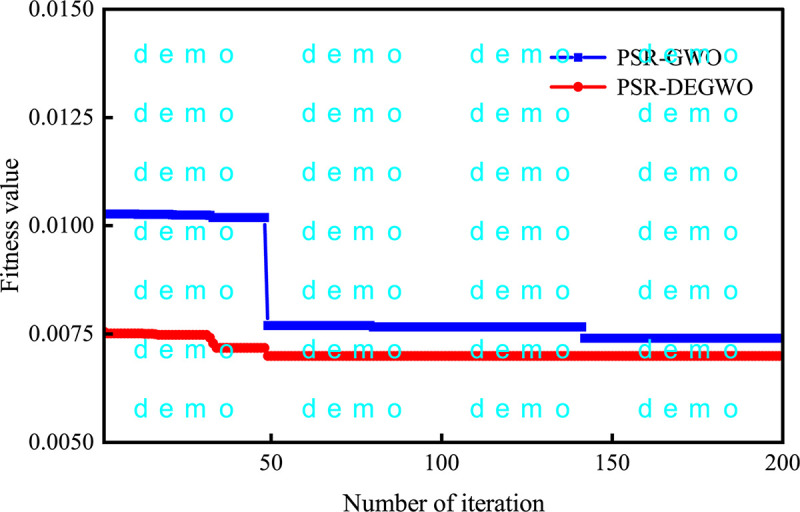
The fitness curves of the GWO and DEGWO model (chaotic) of the PL13-2.

**Fig 37:**
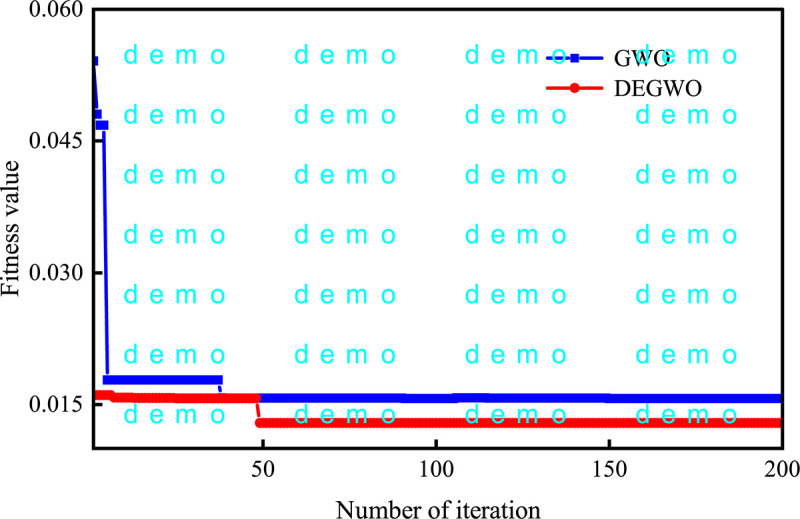
The fitness curves of the GWO and DEGWO model (conventional) of the PL13-3.

**Fig 38:**
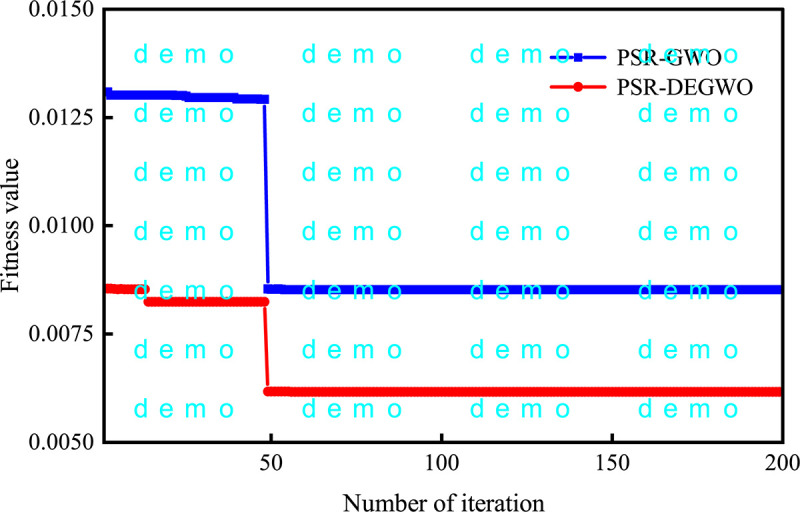
The fitness curves of the GWO and DEGWO model (chaotic) of the PL13-4.

The fitness curves of the GWO and DEGWO algorithm are shown in Figs 35–38. For the conventional model with dam deformation influencing factors as input variables, the optimal parameters [*C*, *γ*] of SVM obtained by the DEGWO algorithm are [4.6616, 0.0100] (PL13-2) and [4.5948, 24.2515] (PL13-3). For the chaotic model with the reconstructed observed deformation phase space as the input variable, the optimal parameters [*C*, *γ*] of the SVM obtained by the DEGWO algorithm are [6.0011, 0.0100] (PL13-2) and [24.2515, 24.2515] (PL13-3).

It can be seen from Figs 35–38, whether it is a conventional model with dam deformation influencing factors as input variables or a chaotic model with reconstructed observation displacement data series phase space as the input variable, the DEGWO algorithm reduces the number of iterations and can find the solution closest to the best goal faster. The main explanation is that the DEGWO algorithm enriches the diversity of the initial population and improves the global search capability, thereby accelerating the convergence speed and convergence accuracy.

### 4.3. Results

For the PSR-SVM-DEGWO-based dam observation displacement prediction model, the relevant information is introduced as follows. The SVM is at the heart of this innovative combination model. The input variable is the reconstructed phase space of the measured displacement data sequence, and the DEGWO algorithm is used to realize the parameter optimization of SVM. In addition, the kernel function of SVM is a radial basis function.

In order to better analyze the predictive performance of the PSR-SVM-DEGWO model, the PSR-SVM and PSR-SVM-GWO models that take the reconstructed phase space of the observed displacement data sequence as input variables are established respectively. In addition, the SVM, SVM-GWO and SVM-DEGWO models with the factors affecting dam deformation as input variables are established to explore the influence of different input variables on the accuracy of model prediction. T-test is adopted to test whether there is a significant difference between the existing method and the proposed PSR-SVM-DEGWO method. The *h* represents whether the hypothesis is accepted at the significance level. When *h* = 1, it means that the two sets of data compared with each other have significant differences. At this time, the comparison between the two algorithms is meaningful. The *p* represents the set standard of significant difference, which is set to 0.05 here. When *p* is less than 0.05, the results have a significant difference. ci represents the data interval with 95% confidence level.

**Fig 39:**
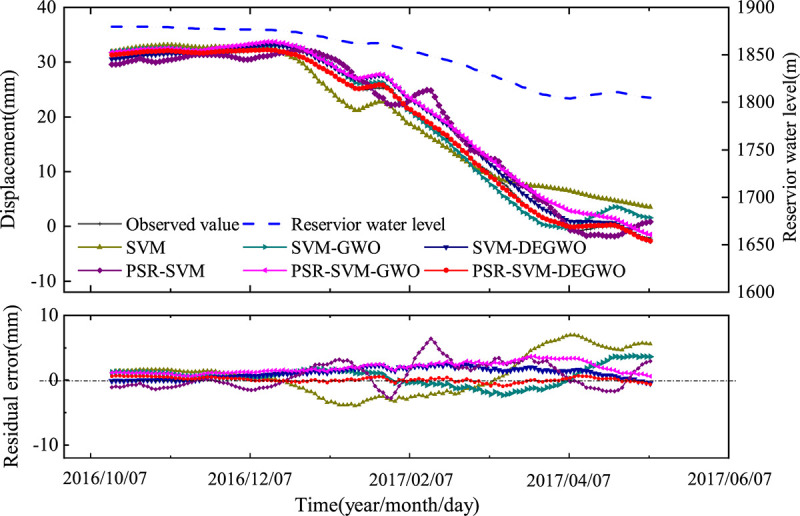
The predicted results of six models at PL13-2.

For the measuring point PL13-2, the prediction performance of the SVM, SVM-GWO, SVM-DEGWO, PSR-SVM, PSR-SVM-GWO and PSR-SVM-DEGWO models are shown in Table 3 and Fig 39. From Table 3, we can see that when the influencing factors of dam deformation are used as the input variables of the model, the square correlation coefficient (R^2^) is ranked from large to small as SVM-DEGWO model> SVM-GWO model> SVM model, mean absolute percentage error (MAPE) is ranked from small to large SVM-DEGWO model< SVM model< SVM-GWO model, and mean square error (MSE) is ranked from small to large SVM-DEGWO model< SVM-GWO model< SVM model. The SVM-DEGWO model has the largest R^2^ of 0.9961, and the smallest MAPE and MSE are 0.3070 and 1.9001, respectively. When the reconstructed phase space of the dam deformation time series is used as the input variable of the model, R^2^ is sorted from large to small as PSR-SVM-DEGWO model> PSR-SVM-GWO model> PSR-SVM model. The ranking of MAPE and MSE from small to large is PSR-SVM-DEGWO model <PSR-SVM-GWO model <PSR-SVM model. The PSR-SVM-DEGWO model has the largest R^2^ of 0.9993, and the smallest MAPE and MSE are 0.2491 and 0.1399, respectively.

**Table 3:** Predictive performance of six models at PL13-2.

Prediction model	MSE	MAPE	R^2^	T-test		
				*h*	*p*	*ci*
**SVM**	10.6873	3.3850	0.9527	1	0.0038	[0.2580,1.3298]
**SVM-GWO**	2.4526	2.0059	0.9873	1	4.2714e-04	[0.2066,0.7158]
**SVM-DEGWO**	1.9001	0.3070	0.9961	1	1.6751e-31	[0.8649,1.1645]
**PSR-SVM**	4.4876	1.1782	0.9768	1	1.0303e-04	[0.3414,1.0226]
**PSR-SVM-GWO**	4.2542	0.7015	0.9971	1	1.1217e-64	[1.6242,1.9321]
**PSR-SVM-DEGWO**	0.1399	0.2491	0.9993	---	---	---

The t-test is used to test whether there are significant differences between the calculation results of the other five algorithms and the results of the proposed PSR-SVM-DEGWO algorithm. It can be seen from Table 3 that all the results meet the condition *h* = 1 and *p*<0.05, which represents the existence of significant differences.

As shown in Fig 39, the deformation shows obvious periodic regular changes, but compared to the conventional arch dam below 200m, the deformation fluctuation range of Jinping I super high arch dam is much larger. SVM, SVM-GWO, SVM-DEGWO, PSR-SVM, PSR-SVM-GWO and PSR-SVM-DEGWO models can effectively predict the trend of dam displacement. And the PSR-SVM-DEGWO model has the highest prediction accuracy and the smallest fluctuation range of the prediction error. For the conventional model, the deviation between the predicted value of the model and the observed value is larger than the deviation of the PSR models.

**Fig 40:**
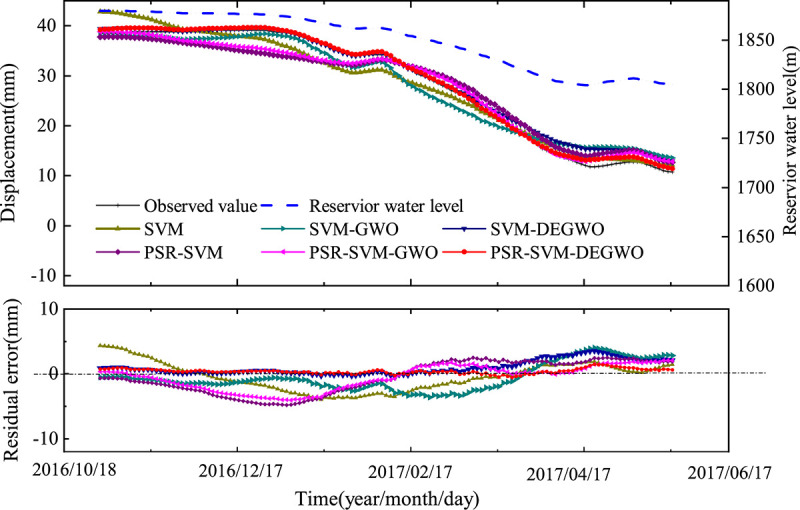
The predicted results of six models at PL13-3.

For the measuring point PL13-3, the prediction performance of the SVM, SVM-GWO, SVM-DEGWO, PSR-SVM, PSR-SVM-GWO and PSR-SVM-DEGWO models are shown in Table 4 and Fig 40. From Table 4, we can see that when the influencing factors of dam deformation are used as the input variables of the model, the square correlation coefficient (R^2^) is also ranked from large to small as SVM-DEGWO model> SVM-GWO model> SVM model, mean absolute percentage error (MAPE) is ranked from small to large SVM model< SVM-DEGWO model< SVM-GWO model, and mean square error (MSE) is ranked from small to large SVM-DEGWO model< SVM-GWO model< SVM model. The SVM-DEGWO model has the largest R^2^ of 0.9971, and the smallest MAPE and MSE are 0.1381 and 2.0937, respectively. When the reconstructed phase space of the dam deformation time series is used as the input variable of the model, R^2^ is sorted from large to small as PSR-SVM-DEGWO model> PSR-SVM-GWO model> PSR-SVM model. The ranking of MAPE and MSE from small to large is PSR-SVM-DEGWO model <PSR-SVM-GWO model <PSR-SVM model. The PSR-SVM-DEGWO model has the largest R^2^ of 0.9987, and the smallest MAPE and MSE are 0.0485 and 0.3347, respectively. The t-test is used to test whether there are significant differences between the calculation results of the other five algorithms and the results of the proposed PSR-SVM-DEGWO algorithm. It can be seen from Table 4 that all the results meet the condition *h* = 1 and *p*<0.05, which represents the existence of significant differences.

**Table 4:** Predictive performance of six models at PL13-3.

Prediction model	MSE	MAPE	R^2^	T-test		
				*h*	*p*	*ci*
**SVM**	5.0227	0.1101	0.9571	1	1.6905e-05	[-1.2225, -0.4646]
**SVM-GWO**	4.8053	0.1846	0.9741	1	2.6575e-07	[-1.3598, -0.6214]
**SVM-DEGWO**	2.0937	0.1381	0.9971	1	4.9553e-08	[0.3598,0.7483]
**PSR-SVM**	5.0054	0.2102	0.9765	1	4.2302e-05	[-1.3451, -0.4815]
**PSR-SVM-GWO**	3.9390	0.1054	0.9815	1	5.7399e-09	[-1.3464, -0.6829]
**PSR-SVM-DEGWO**	0.3347	0.0485	0.9987	---	---	---

As shown in Fig 40, the deformation shows obvious periodic regular changes, but compared to the conventional arch dam below 200m, the deformation fluctuation range of Jinping I super high arch dam is much larger. SVM, SVM-GWO, SVM-DEGWO, PSR-SVM, PSR-SVM-GWO and PSR-SVM-DEGWO models can effectively predict the trend of dam displacement. And the PSR-SVM-DEGWO model has the highest prediction accuracy and the smallest fluctuation range of the prediction error. For the conventional model, the deviation between the predicted value of the model and the observed value is larger than the deviation of the PSR models.

The calculation results show: ①The applicability of the GWO optimized SVM algorithm in dam deformation prediction; ②The DEGWO algorithm proposed in this paper has more outstanding optimization ability in optimizing SVM parameters than the conventional GWO; ③For ultra-high arch dams, the PSR model with the reconstructed phase space as the input variable has higher prediction accuracy and smaller prediction error than its corresponding conventional model with dam deformation influence factors as input variables.

In general, the SVM, SVM-GWO, and SVM-DEGWO models can better predict the change trend of dam deformation. Regardless of whether it is a conventional model with the dam deformation influence factors as input variables or the PSR model with the reconstructed observation displacement data series phase space as the input variable, the prediction accuracy of all the model can meet the engineering requirements, but the prediction accuracy of the PSR model is higher. The calculation results also prove the applicability of the GWO algorithm in the field of dam deformation prediction and the more prominent optimization ability of DEGWO compared to GWO. The t-test results show that the calculation results of the other five algorithms are significantly different from the results of the proposed PSR-SVM-DEGWO algorithm. The result of t test also shows that the algorithm comparison is meaningful, no matter it is for the measuring point PL13-2 or PL13-3.

## 5. Conclusions

This research proposes an innovative model combining chaos theory, support vector machine, difference algorithm and gray wolf algorithm, namely the PSR-SVM-DEGWO model, to predict dam deformation. And taking the measured displacement data of the Jinping I super high arch dam as examples, the prediction effect of the PSR-SVM-DEGWO model is compared and verified. The main conclusions are as follows.

1. As the correlation dimension of the deformation time series tends to be saturated (*D*_2-2_ = 1.2043, D_2-3_ = 1.1217), the largest Lyapunov exponent (*λ*_max-2_ = 0.0191, *λ*_max-3_ = 0.0108) is greater than 0 and the Kolmogorov entropy estimate (*K*_2-2_ = 0.0043, *K*_2-3_ = 0.0044) is a finite positive value, it can be seen that there is chaos in the deformation observation data of the dam.
2. The optimization performance of the DEGWO algorithm is superior to that of the GWO algorithm. Using the DE algorithm to ensure the initial population diversity can effectively improve the grey wolf optimization algorithm’s ability to find high-quality solutions. Simulation tests show that the convergence speed of the DEGWO algorithm is faster and the convergence accuracy is higher.
3. It is verified by the example of Jinping I super high arch dam that SVM, SVM-GWO and SVM-DEGWO models can effectively predict the dam deformation trend, but the SVM-DEGWO model has the best prediction performance, which is reflected in the higher accuracy of the model prediction.
4. The predictive performance of the PSR-SVM, PSR-SVM-GWO and PSR-SVM-DEGWO models with the reconstructed observation data sequence phase space as the input variable is superior to that of the corresponding SVM, SVM-GWO and SVM-DEGWO conventional models with deformation influence factors as input variables. When the conventional model predicts the deformation of an ultra-high arch dam, although the accuracy meets the requirements, the predicted value will gradually deviate from the measured value as time goes by. On the contrary, it is difficult to observe such large deviations in models that adopt the reconstructed phase space of the observation data sequence as the input variable. Among all the models calculated in this paper, the PSR-SVM-DEGWO model has the best prediction performance.
